# A protocol for monitoring plant responses to changing nitrogen deposition regimes in Alberta bogs

**DOI:** 10.1007/s10661-020-08645-z

**Published:** 2020-11-02

**Authors:** Dale H. Vitt, Melissa House, Samantha Kitchen, R. Kelman Wieder

**Affiliations:** 1 School of Biological Sciences, Southern Illinois University, Carbondale, IL 62901 USA; 2 Department Biology, Villanova University, Villanova, PA 19085 USA; 3 Center for Biodiversity and Ecosystem Stewardship, Villanova University, Villanova, PA 19085 USA; 4 Faculty of Science and Technology, Athabasca University, Athabasca, Alberta Canada

**Keywords:** Allometric equation, Atmospheric deposition, Bog, Boreal, Nitrogen, Peatland, Oilsands, *Sphagnum*

## Abstract

Bogs are nutrient poor, acidic ecosystems that receive their water and nutrients entirely from precipitation (= ombrogenous) and as a result are sensitive to nutrient loading from atmospheric sources. Bogs occur frequently on the northern Alberta landscape, estimated to cover 6% of the Athabasca Oil Sands Area. As a result of oil sand extraction and processing, emissions of nitrogen (N) and sulfur (S) to the atmosphere have led to increasing N and S deposition that have the potential to alter the structure and function of these traditionally nutrient-poor ecosystems. At present, no detailed protocol is available for monitoring potential change of these sensitive ecosystems. We propose a user-friendly protocol that will monitor potential plant and lichen responses to future environmental inputs of nutrients and provide a structured means for collecting annual data. The protocol centers on measurement of five key plant/lichen attributes, including changes in (1) plant abundances, (2) dominant shrub annual growth and primary production, (3) lichen health estimated through chlorophyll/phaeophytin concentrations, (4) *Sphagnum* annual growth and production, and (5) annual growth of the dominant tree species (*Picea mariana*). We placed five permanent plots in each of six bogs located at different distances from the center of oil sand extraction and sampled these for 2 years (2018 and 2019). We compared line intercept with point intercept plant assessments using NMDS ordination, concluding that both methods provide comparable data. These data indicated that each of our six bog sites differ in key species abundances. Structural differences were apparent for the six sites between years. These differences were mostly driven by changes in *Vaccinium oxycoccos*, not the dominant shrubs. We developed allometric growth equations for the dominant two shrubs (*Rhododendron groenlandicum* and *Chamaedaphne calyculata*). Equations developed for each of the six sites produced growth values that were not different from one another nor from one developed using data from all sites. Annual growth of *R. groenlandicum* differed between sites, but not years, whereas growth of *C. calyculata* differed between the 2 years with more growth in 2018 compared with 2019. In comparison, *Sphagnum* plant density and stem bulk density both had strong site differences, with stem mass density higher in 2019. When combined, annual production of *S. fuscum* was greater in 2019 at three sites and not different at three of the sites. Chlorophyll and phaeophytin concentrations from the epiphytic lichen *Evernia mesomorpha* also differed between sites and years. This protocol for field assessments of five key plant/lichen response variables indicated that both site and year are factors that must be accounted for in future assessments. A portion of the site variation was related to patterns of N and S deposition.

**Electronic supplementary material:**

The online version of this article (10.1007/s10661-020-08645-z) contains supplementary material, which is available to authorized users.

## Introduction

Bogs are *Sphagnum*-dominated, acidic, nutrient poor, peat-accumulating ecosystems found in subarctic, boreal, and cool-temperate regions, mainly in the northern hemisphere (Vitt 2006). In western Canada, they cover 365,157 km^2^ (or about 20%) of the land surface in Alberta, Saskatchewan, and Manitoba (Vitt et al. 2000). Hydrologically, bogs are ombrogenous, receiving water and nutrient inputs only from atmospheric sources and have naturally low nutrient and base cation levels. In pristine areas, nutrient inputs are completely absorbed and utilized in growth by the plants and lichens occupying the bog ecosystem (Wieder et al. 2019). Species richness is low, and plant communities exhibit high species similarity across sites (Vitt et al. 1995a). All bogs in continental Canada have a canopy dominated by one tree species, *Picea mariana*, a shrub layer of 5–6 species of Ericaceae and dominated by either *Rhododendron (Ledum) groenlandicum* or *Chamaedaphne calyculata*, and a hummocky ground layer mostly dominated by one species of peat moss - *Sphagnum fuscum* (Vitt et al. 1995a). Fruticose lichens (especially *Evernia mesomorpha* and *Usnea* spp*.*) are abundant on tree branches (Vitt 2006). These species-poor plant communities are sensitive to environmental changes and, owing to their ombrogenous water source, are especially sensitive to nutrient loading from atmospheric sources (Wieder et al. 2019).

Research in European bogs has shown that increasing atmospheric nitrogen (N) deposition leads to changes in ecosystem structure and function, including altered growth and species composition of *Sphagnum* mosses, increase in vascular plant abundances, increased N concentrations and quantities in peat, and increased NH_4_^+^-N, NO_3_^−^-N, and/or dissolved inorganic N concentrations (DIN) in bog porewaters (e.g., Berendse et al. 2001; Bragazza and Limpens 2004; Bragazza et al. 2005; Gunnarsson and Rydin 2000; Gunnarsson et al. 2004; Hoosbeek et al. 2002; Lamers et al. 2000; Limpens and Berendse 2003; Limpens et al. 2003; Wiedermann et al. 2008). In eastern North America, at Mer Bleue bog in eastern Ontario, after 5 years of N fertilization, Bubier et al. (2007) reported an increase in vascular plant cover and decease in *Sphagnum* abundance. However, all of these studies were done in areas where ambient levels of N deposition have been significantly higher than those found in western Canada. In addition, many studies have shown that lichens, especially fruticose growth forms, are among the most sensitive components of vegetation to increasing N inputs (e.g., Gilbert 1986; Van Dobben et al. 2001).

The Oil Sands Administrative Area in northern Alberta occupies 140,329 km^2^ with bogs covering 6% of the area (Wieder et al. 2016b). Atmospheric deposition of N is low in the region (< 1 kg N ha^−1^ year^−1^; Hember 2018; Wieder et al. 2016a); however, oil sand development north of Fort McMurray, Alberta, has led to increasing N deposition with values as high as 17 kg N ha^−1^ year^−1^ (Fenn et al. 2015; Wieder et al. 2016a). These high values of atmospherically deposited N have the potential to alter the structure and function of these traditionally nutrient-poor ecosystems; however, no detailed protocol is available for monitoring potential change of these sensitive ecosystems. Although the Alberta Biodiversity Monitoring Institute has a province-wide program to track Alberta’s wildlife and their habitats, and tracks the health of many species (ABMI 2017), the program is not designed to assess ecosystem changes on an annual basis. Furthermore, bogs are only a small part of their broad program. We are aware of only two studies that aimed to track the change of vegetation through time in wetland plant communities, and both of these determined vegetation change after restoration of bogs recovering from horticultural peat mining in eastern Canada (Poulin et al. 2013; Rochefort et al. 2013).

Previous research at a bog near Mariana Lakes, Alberta, experimentally added N over a 5-year time period (at levels up to 25 kg N ha^−1^ year^--1^, as NH_4_NO_3_ in simulated rainfall) At the higher levels, *Sphagnum fuscum* net primary production (NPP) was inhibited, dominant shrub and *Picea mariana* aboveground NPP were stimulated, with increased root biomass and production, and *Sphagnum* species relative abundance changed. Higher N deposition also increased the abundance of *Rhododendron groenlandicum* and *Andromeda polifolia* and to vascular plants in general, with an increase in shrub leaf N concentrations in *Andromeda polifolia*, *Chamaedaphne calyculata*, *Vaccinium oxycoccos* (= *Oxycoccus microcarpus*), *V. vitis-idaea*, and *Picea mariana* (Wieder et al. 2019). Of fundamental importance to the N cycle in bogs, this research showed that biological N_2_-fixation greatly exceeded atmospheric N deposition as a source of new N inputs to the bog; however, an increase in N addition stimulated N_2_-fixation at deposition < 3.1 kg N ha^−1^ year^−1^ and progressively inhibited N_2_-fixation as N deposition increased above this level.

These unique attributes of bogs (e.g., ombrotrophy) and their responses to environmental conditions from atmospheric deposition make them target ecosystems for monitoring changes in atmospheric inputs of nutrients. In particular, N inputs are a primary stressor of concern in many regions affected by anthropogenic disturbances (Galloway et al. 2008; Kanakidou et al. 2016). Here we propose a multi-faceted user-friendly approach designed to monitor plant/lichen responses to changing atmospheric deposition regimes in bogs utilizing five key components of bog ecosystems. Indicators were selected based on the following criteria: (1) responsive to stressors, (2) cost-effective, (3) repeatable, and (4) have species and community features that are easy to quantify and identify by field personnel with a moderate level of taxonomic expertise. Although species in the genus *Sphagnum* are thought to be taxonomically difficult, only three species commonly occur in Alberta bogs, and these are easily differentiated by color alone. Data for all of these indicators are easily collected in the field and analyzed in the laboratory at minimal cost. Our primary objective was to develop a monitoring protocol for assessing key parameters of bog ecosystems that are appropriate for long-term permanent monitoring stations. Our objectives were centered around six questions. (1) For the plant community—over a multi-year time frame with different field personnel, is a line transect method of plant abundance assessment appropriate for monitoring? (2) Can a line transect with numerous contiguous points of measurement be reduced to a more limited point transect method? (3) For plant growth and production—can we develop an easy, non-destructive, field-based method for assessing plant annual growth and production utilizing the 1–2 dominant shrub species present at each site? (4) Using only one lichen species (*Evernia mesomorpha*), can we develop a suitable technique for using lichen chlorophyll/phaeophytin ratios as an indicator of lichen health? (5) Using cranked wires how variable is annual growth and NPP of *Sphagnum*? (6) Can we use leader length of *Picea mariana* to assess variability in growth of this species? Here we assess the outcomes of these protocols over a 2-year time period for a series of six bog sites in northeastern Alberta, Canada, and provide evidence of whether the variability in responses can be related to growing season N and S deposition from oil sands activities.

## Materials and methods

### Site selection

Peat-forming wetlands (bogs and fens) are a major feature of Alberta’s landscape (Vitt et al. 1995b) and cover 6% and 21%, respectively, of the 140,329 km^2^ Oil Sands Administrative Area in northeastern Alberta (Wieder et al. 2016a). We chose six bog sites (Table 1), centered on the Syncrude/Suncor Canadian oil sands active open-pit mines and refineries, north of Fort McMurray, Alberta. Sites ranged from 12 to 77 km from oil sands refineries. All bogs have attributes described by Belland and Vitt (1995) and are characterized as follows: a tree canopy of mature (from 69 to 82 years old (to 2019) *Picea mariana*, a shrub layer of *Rhododendron groenlandicum*, *Chamaedaphne calyculata*, *Vaccinium oxycoccos*, *V. vitis-idaea*, and *Kalmia polifolia*, a forb layer of *Rubus chamaemorus* and *Maianthemum* (*Smilacina*) *trifolium*, and a ground layer of 100% mosses dominated by *Sphagnum fuscum*, with occasional hummocks of *Pleurozium schreberi* and *S. capillifolium* and lawns of *S. angustifolium*. The lichen, *Evernia mesomorpha*, is abundant on tree branches. At the top of the bog water table, porewater mean pH at these six bog sites ranged from 3.81 to 4.69 and mean reduced (corrected for pH) electrical conductivity ranged from 18 to 131 μS cm^−1^. Regional climate is characterized by 164–221 mm growing season precipitation, a mean July daily temperature of 14.5–15.1 °C, and a growing season aridity index (PET/Precipitation) of 1.7–2.6 (Wieder et al. 2016b).

**Table 1:** Locations of six bog sites used in this study. Distance refers to distance from midpoint between Syncrude Canada and Suncor refinery stacks (km)

Site	Latitude	Longitude	Distance
JPH4	57° 06′ 45″ N	111° 25′ 23″ W	12
McKay	57° 13′ 41″ N	111° 42′ 11″ W	24
Kearl	57° 19′ 04″ N	111° 13′ 07″ W	32
McMurray	56° 37′ 37 ″ N	111° 11′ 44″ W	49
Anzac	56° 28′ 08 ″ N	111° 02′ 34″ W	69
Horse Creek	56° 19′ 46 ″ N	111° 35′ 22″ W	77

### Sampling design

At each of the six bog sites, we used a restricted random design and placed five plots approximately 30–60 m apart. Each plot contained a 300-cm-long transect placed in a large hummock of *Sphagnum fuscum*, two 1-m^2^ shrub abundance plots, a meandering transect for collecting biomass of *Evernia mesomorpha*, and 10 randomly chosen *Picea mariana* trees for leader length measurement. All bogs had an open canopy, and individual trees from 1 to 2 m tall were chosen and marked with aluminum tags.

### Plant community composition

Reviews of sampling methods for plant abundances are found in McCune and Grace (2002) and Phillips (1959). We considered several alternatives for sampling. Releve’s (Braun-Blanquet 1932) are based on lists of species with visual estimation of cover within an area of homogenous vegetation. Cover estimates are potentially biased by different observers and require a thorough knowledge of the regional flora. Comparatively, the point-observation methods yield an estimate of cover of each species in a standardized plot (Clements 1905). These plots may be fixed contiguously along a transect, randomly placed along a transect, or restricted to transect segments, and often have nested subplots for different sized vegetation units. Both methods suffer from over-estimates in the cover of rare species and yield imprecise estimates of the more abundant species, especially since repeated measurements are usually done on the same plots by different observers. Another method that provides repeatable data is the line-intercept method (Canfield 1941) that uses percent cover as a proportion of the total length of each species along a line in comparison with the total length of the line. Although the line-intercept method provides a rich set of data, it suffers from being relatively tedious and time-consuming in data collection. The point-intercept method (Goodall 1953) provides a data set of frequency values for points placed along a line; it takes less time to complete but may provide a less detailed data set that may not include the full suite of species found along the transect. Points can be randomly chosen or systematically placed along the line. Both line-intercept and point-intercept methods use absolute frequency, or the proportion of occurrences of a species relative to the number of sample units taken along the line transect.

We used a 300-cm-long line transect, with the transect usually divided into two portions in order to fit on a single large *Sphagnum* hummock, to characterize vascular plant, bryophyte, and lichen community composition. *Sphagnum* hummocks were chosen from among those dominated by *S. fuscum* (or *S. capillifolium* in one case) without evidence of disturbance. For each transect, permanent, plastic survey stakes were placed at the ends and the transect delineated by a cloth measuring tape held straight and anchored at ground level by the stakes at each end of the transect. Species presence was recorded at every centimeter along the 300-cm line. A ruler, kept vertically straight, was used to assist in determining whether a living vascular plant, bryophyte, or ground lichen was present or absent at a given point. Data were not transformed and were analyzed as absolute frequency (number of hits/300). In order to determine if point-intercept data would mirror the data from the 300 cm line transect, frequency data from 30 and 60 evenly spaced points along the transect were extracted and compared with the 300-point line transect.

### Lichen chlorophyll and phaeophytin

Approximately 30–40 thalli of the lichen *Evernia mesomorpha* were collected from each plot in paper bags. Lichen thalli were dried at room temperature and kept at − 20 °C until chlorophyll and phaeophytin analysis. Prior to analysis, 20 mg of lichen tissue (tissue taken from the apical parts of the thalli) were taken from 10 randomly selected thalli for each plot, for a total of 50 samples per site. To extract chlorophyll and determine percent phaeophytin, samples were extracted using dimethyl sulfoxide (DMSO). Acetone has also been used for this extraction (Arnon 1949; Barnes et al. 1992). The DMSO method can be used with fresh, not ground, tissue and has been found to be comparable to the acetone method (Ronen and Galun 1984; Barnes et al. 1992; Tait and Hik 2003). Using test tubes, 5 mL of dimethyl sulfoxide (DMSO) was added to 10 samples of lichen tissue at a time to extract chlorophyll (Ronen and Galun 1984). After incubating the 10 samples for 45 min at 60 °C, another 5 mL of DMSO were added to each sample. Samples were transferred to a cuvette and analyzed on a spectrophotometer at 415, 433, 645, and 665 nm to measure optical density (OD). Chlorophyll *a* and *b* concentrations were calculated using the equation (Arnon 1949; Tait and Hik 2003):

$$ \mathrm{mg}\ \mathrm{chlorophyll}\ {\mathrm{g}}^{-1}\mathrm{air}\ \mathrm{dry}\ \mathrm{weight}=\frac{\left(20.2\ast \left({\mathrm{OD}}_{645}\right)+8.02\ast \left({\mathrm{OD}}_{665}\right)\right)\ast 10\ \mathrm{mL}}{1000\ \mathrm{mL}\ast \mathrm{g}\ \mathrm{air}-\mathrm{dry}\ \mathrm{weight}} $$

To determine chlorophyll degradation and the percent of phaeophytin, we used the ratio of OD at 433:415 or the ratio of chlorophyll *a* to phaeophytin *a* pigments. Using an OD_433_:OD_415_ curve (Ronen and Galun 1984), we used the following equation to calculate the chlorophyll *a* to phaeophytin *a* ratio:

$$ \%\mathrm{phaeophytin}\ a=-104.8\ln \left(\mathrm{OD}433:\mathrm{OD}415\right)+39.715 $$

### Aboveground shrub growth (MPS) and aboveground NPP

We know of no information on predicting net primary production for bog shrubs using nondestructive sampling and allometric equations except for that in Wieder et al. (2019). Connolly and Grigal (1983) in Minnesota and He et al. (2018) in Alberta published allometric equations for fen shrub biomass, but none of the species utilized is found in bogs. To determine annual growth and aboveground net primary production (NPP) for the dominant two shrubs at each site, we destructively sampled new growth segments (shoots with leaves) from *Rhododendron groenlandicum* and *Chamaedaphne calyculata*. In July 2019, we collected 20 new growth segments (new growth from the most recent bud scar) of the two dominant species at each of five plots—outside of our 1-m^2^ quadrats—for the six sites to develop allometric equations as a means for non-destructive estimation of NPP*.* After drying the growth segments at 55 °C for 1 week, we quantified dry mass and determined the mass per new growth segment (MPS). For each new growth segment, we measured the shoot length (mm), the number of leaves, and the length and maximum width (mm) of each leaf. During the drying process, *R. groenlandicum* leaves slightly curled, thus affecting leaf width measurements. Using measurements of living *R. groenlandicum* leaves from our 2018 and 2019 field data, we were able to predict the width and area of dried *R. groenlandicum* leaves:

$$ \mathrm{LA}=0.2798\times {\mathrm{LL}}^{1.8156};{R}^2=0.87 $$

where LA is the leaf area (mm^2^) and LL is the leaf length (mm),

$$ \mathrm{LW}=\frac{4\times LA}{LL\times \pi } $$

and LW is the leaf width (mm), LA is the leaf area (mm^2^), and LL is the leaf length (mm). Adapted from Wieder et al. (2019), we developed allometric growth equations that gave the best linear fit to determine MPS (mg segment^−1^) for *R. groenlandicum* and *C. calyculata* at each site (Table 2). We used the modeled MPS and density of shoots for each species to calculate NPP.

**Table 2:** Best linear fit parameters and *r*^2^ values for equations relating mg per new growth segment (MPS; g dry mass per segment) to average leaf length (ALL, mm), average leaf width (ALW, mm), average segment length (ASL, mm), and average number of leaves per segment (ALPS; # per segment), using the following general equation: $\mathrm{MPS}=a\times \sqrt{\Big(0.5\times \mathrm{ALL}\times 0.5\times \mathrm{ALW}\times \pi \times \mathrm{ASL}\times \mathrm{ALPS}}\Big)+b$.

		Parameter estimates		
	Site	*a*	*b*	*R*^2^
*R. groenlandicum*	JPH4	0.0011	0.0044	0.86
	McKay	0.0009	0.0134	0.85
	Kearl	0.0012	− 0.0092	0.86
	McMurray	0.0012	− 0.0047	0.88
	Anzac	0.0009	0.0283	0.80
	Horse Creek	0.0012	− 0.0118	0.90
	All sites combined	0.0010	0.0057	0.85
*C. calyculata*	JPH4	0.0005	− 0.0065	0.94
	McKay	0.0004	0.0012	0.91
	Kearl			
	McMurray	0.0005	− 0.0027	0.89
	Anzac	0.0005	− 0.0038	0.91
	Horse Creek	0.0005	− 0.0107	0.91
	All sites combined	0.0005	− 0.0032	0.91

Net primary production (NPP) of *Sphagnum fuscum* is the product of linear growth of stems (cm year^−1^) and stem mass density (SMD), with SMD defined as the mass of 1-cm lengths of *Sphagnum* stems beneath the capitula per square meter. We used the cranked wire method (Clymo 1970; Vitt 2007) to measure linear growth of *Sphagnum fuscum*. We set 30 cranked wires in each of the five plots, with all wires placed in hummocks dominated by *S. fuscum*. Annually, we set cranked wires in early May (after ice thawed from the upper peat) and remeasured them in late September or early October. To measure SMD, we collected surface cores (6.5 cm diam.) from *S. fuscum* hummocks in each plot in early July of each year. From each core, all capitula were removed and counted to determine *S. fuscum* plant density (plants m^2^). Seventy stems of *S. fuscum* were selected, cut into 2-cm lengths, and weighed after drying at 55 °C for 5 days. SMD was calculated as the average mass of a 1-cm stem of an individual *Sphagnum* plant multiplied by plant density.

### Leader length of *Picea mariana*

Bogs of the region are dominated by a single tree species *Picea mariana* (black spruce). Stand dynamics of this species have been examined by Wieder et al. (2009). Mature bogs of the region are mostly less than 100 years old, and stand replacement is largely by wildfire. In July 2019, ten *Picea mariana* trees were selected along a transect at each plot. Leader length (or terminal leader extension) for one growing season was quantified by measuring from the second most recent bud scale to the most recent bud scale, thus our data for leader length are for the 2018 growing season.

### Data analyses

Analyses for plant community composition were based on absolute frequency data from each plot from each site. To determine differences between sites and changes in plant community composition between years, we used Bray-Curtis similarity nonmetric multidimensional scaling (NMDS) in Primer 7 (Clarke and Gorley 2006) with 100 restarts and no pretreatment of the data. A 2-way PERMANOVA was used to determine differences between years and sites (fixed factors). For factors with significant differences, post hoc pair-wise tests were used to determine which sites and years differed.

To determine whether point-intercept data could replace our line-transect method, we used subsets of data from points located every 5 cm (60 points along the transect) and at every 10 cm (30 points along the transect). Within each year (2018 and 2019), we used a one-way PERMANOVA to test whether the subsets differed from the entire transect (30, 60, and 300 points [fixed effect]).

Statistical analyses for *Evernia mesomorpha* chlorophyll *a* and phaeophytin *a* concentrations, aboveground *Rhododendron groenlandicum* and *Chamaedaphne calyculata* abundance, MPS and NPP, and *Picea mariana* leader length were performed in JMP (JMP v. Pro 14.3.0). *Picea mariana* leader length was tested between sites using a one-way nested (subsamples nested within plots) analysis of variance (ANOVA) design and Tukey’s pairwise post hoc comparisons when data passed the Shapiro-Wilks test for normality (*p* < 0.05 failure and visual inspection of residuals). *Evernia mesomorpha* chlorophyll and phaeophytin were analyzed using a two-way ANOVA (site, year as fixed effects) with subsamples nested within plot (random effect) and with Tukey’s pairwise post hoc comparisons. Shrub abundance, MPS, and NPP were analyzed using a two-way ANOVA (site, year; fixed effects) with subplots nested within plots (random effect) and Tukey’s pairwise post hoc comparisons when data passed the Shapiro-Wilks test for normality (*p* < 0.05 failure and visual inspection of residuals). Non-normal data were either square-root (2018 vs. 2019 *Rhododendron groenlandicum* predicted NPP), cube-root (*Evernia mesomorpha* chlorophyll concentrations and percent phaeophytin, *Rhododendron groenlandicum* predicted MPS vs. dry weight), or log-transformed (2018 vs. 2019 *Rhododendron groenlandicum* predicted MPS*, Picea mariana* leader length) to improve homoscedasticity. To compare the site-specific MPS algorithms with the overall algorithm, ANCOVA was used to test for differences in slopes (SAS v.9.4).

*Sphagnum fuscum* plant density and stem mass density were assessed with a two-way ANOVA, with year and site as the main effects; a posteriori means comparisons were carried out using Tukey’s HSD. The multiple cranked wires were treated as subsamples within each of the five replicate plots at each site. Thus, both linear growth and annual NPP (linear growth × stem mass density) were analyzed with a nested ANOVA; a posteriori means comparisons were carried out using Tukey’s HSD. For linear growth and NPP, we report means ± standard errors of the plots (*n* = 5 per site), not of the individual cranked wires.

We used atmospheric deposition data for 2018–2019 growing seasons from R. K. Wieder (Villanova University, unpublished data) for JPH4, McKay, McMurray, Anzac, Horse Creek, and Kearl. Deposition data from the six sites were collected using ion exchange resin collectors (Fenn et al. 2002, 2003) with methods described in Wieder et al. 2016b. We examined possible relationships between structural and functional response variables as a function of NH_4_^+^-N, NO_3_^−^-N, DIN, SO_4_^−2^-S, and distance from the midpoint between the Syncrude and Suncor upgrader stacks using linear regression.

## Results

### Plant community composition

#### Species diversity

We recorded a total of 22 species from our plots located on *S. fuscum* hummocks at the six bog sites, 11 were vascular plants, 10 bryophytes (with 4 *Sphagnum* species), and one lichen. About 41% of the species were found at all six sites; similarity for vascular plants was high with eight of the vascular plants found at all sites, but low for bryophytes, with only one species occurring at all sites (Table 3). Mean alpha (plot) diversity was high (15.5), and beta diversity (species turnover between plots) was low (1.42).

**Table 3:** List of species and occurrence (+) at the six bog sites for 2018 and 2019. Nomenclature: *Cladonia = Cladina; Rhododendron (Rhodo.) = Ledum; Maianthemum = Smilacina; Vaccinium oxycoccos = Oxycoccus microcarpus*

Species name	Anzac		Horse Creek		JPH4		Kearl		McKay		McMurray	
	2018	2019	2018	2019	2018	2019	2018	2019	2018	2019	2018	2019
*Andromeda polifolia*	+	+	+	−	−	−	−	−	−	−	−	−
*Aulacomnium palustre*	−	−	−	−	+	+	+	+	−	+	−	−
*Chamaedaphne calyculata*	+	+	+	+	+	+	+	−	+	+	+	+
*Cladonia mitis*	-	−	+	+	−	−	−	−	−	−	−	−
*Dicranum undulatum*	−	−	−	−	−	−	+	+	−	−	−	−
*Drosera rotundifolia*	+	+	+	+	+	+	+	+	+	+	+	+
*Eriophorum vaginatum*	+	+	−	−	+	+	−	−	+	+	+	+
*Kalmia polifolia*	+	+	+	+	+	+	+	+	+	+	+	+
*Mylia anomala*	+	+	+	+	−	−	+	+	+	−	+	+
*Vaccinium oxycoccos*	+	+	+	+	+	+	+	+	+	+	+	+
*Picea mariana*	+	+	+	+	+	+	+	+	+	+	+	+
*Pleurozium schreberi*	−	−	+	+	+	+	+	+	+	+	−	−
*Pohlia nutans*	−	−	-	−	+	+	+	+	+	+	+	+
*Polytrichum strictum*	-	−	−	−	+	+	+	+	+	+	+	+
*Rhodo. groenlandicum*	*+*	+	+	+	+	+	+	+	+	+	+	+
*Rubus chamaemorus*	−	+	+	+	+	+	+	+	+	+	+	+
*Maianthemum trifolium*	+	+	-	−	-	−	+	+	+	+	+	+
*Sphagnum angustifolium*	+	+	-	−	+	+	+	+	-	−	−	−
*Sphagnum capillifolium*	−	−	−	−	+	+	−	−	−	−	−	−
*Sphagnum fuscum*	+	+	+	+	+	+	+	+	+	+	+	+
*Sphagnum magellanicum*	+	+	−	−	+	+	−	−	+	+	−	−
*Vaccinium vitis-idaea*	+	+	+	+	+	+	+	+	+	+	+	+

#### Structural differences among sites and years

**Fig. 1:**
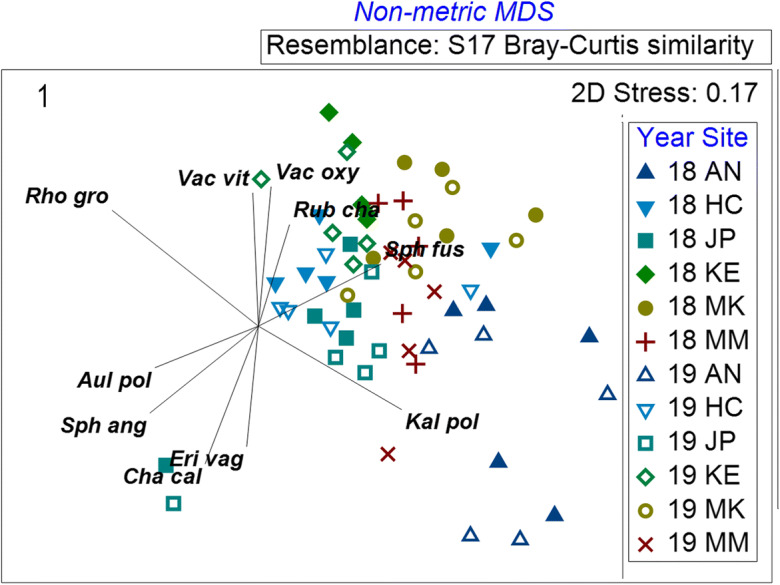
NMDS ordination of plant community composition for 2018 (18) and 2019 (19). Sites are designated by color and shape, and years are distinguished by color fill or + vs. x. Vectors describing species abundance patterns are shown as lines radiating from a common origin, offset to left of center of the ordination (Clarke & Gorley 2006). *AN* = Anzac, *HC* = Horse Creek, *JP* = JPH4, *KE* = Kearl, *MK* = McKay, *MM* = McMurray

The years (2018 and 2019) differed (pseudo-*F* = 9.546, *p* = 0.001), and sites differed (pseudo-*F* = 9.984, *p* = 0.001). The six sites all differed from one another (post hoc pairwise test, *p* < 0.05). Differences between sites are evident on the NMDS ordination (Fig. 1), with individual sites clustered at different locations on the ordination (Kearl plots are positioned to the upper center, McKay to the upper-right, Anzac to the lower right, McMurray center, Horse Creek to the left center, and JPH4 to the center and lower left). Plots at Anzac and two plots at JPH4 are most dissimilar, while plots from the 2 years (2018, 2019) cluster together. Differences in dominant species at the sites are evident on the ordination for a number of species (species vectors in Figs. 1 and 4 and on Online resource 1A-H) including *Chamaedaphne calyculata* (OR1A)—most abundant to the lower right at some Anzac and McMurray plots, *Kalmia polifolia* (OR1B)—abundant to the lower right at other Anzac plots, and *Rhododendron groenlandicum* (OR1C)—more abundant to the upper portion of the ordination at Kearl, McKay, and Horse Creek. Likewise, *Vaccinium oxyxoccos* is more abundant at these latter sites (upper portion of ordination) (Fig. OR1D). *Pohlia nutans* (Fig. OR1E) and *Rubus chamaemorus* (Fig. OR1F) are most abundant at McKay (to the upper right). Two plots at JPH4 were dominated by *Sphagnum angustifolium* (Fig. OR1G), while all others had *S. fuscum* as the dominant moss (Fig. OR1H). When all six sites are considered together, abundance of *Rhododendron groenlandicum* was 1.2 times higher than the combined abundances of *Chamaedaphne calyculata*, *Kalmia polifolia*, and *Andromeda polifolia* (*F* = 35.41, *p* < 0.001).

#### Structural differences between years

**Fig. 2:**
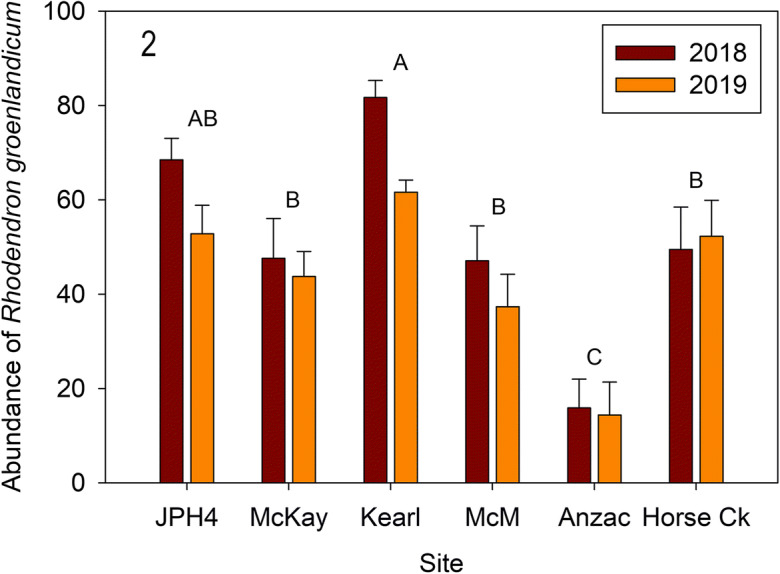
Mean (± S.E.) abundance (as absolute frequency) of *Rhododendron groenlandicum* for 2018 and 2019 along the 300 cm line transect. Result is from a two-way ANOVA for year and site (*p* <0.05), with different letters indicating significantly different values between sites (Tukey’s pairwise post-hoc test at *p* = < 0.05). *McM* = McMurray

**Fig. 3:**
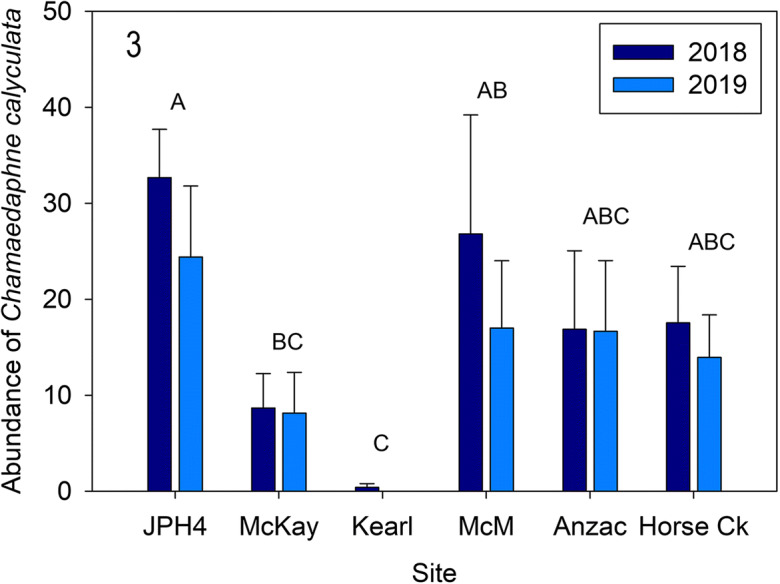
Mean (± S.E.) abundance (as absolute frequency) of *Chamaedaphne calyculata* for 2018 and 2019 along the 300 cm line transect. Different letters indicate significantly different values (*p* < 0.05 Tukey’s test). *McM* = McMurray

Attributes contributing to between year differences include the following: all sites recorded less vascular plant cover in 2019 ranging from 17 (Anzac) to 34% (McMurray) lower vascular plant abundance; these higher 2018 values were largely due to differences in estimates of *Vaccinium oxycoccos* frequency (overall = 51%; ranging from 34 (JPH4) to 52% (Horse Creek and McKay) of the overall dissimilarity between years). Dominant shrub species contributed to the overall dissimilarity—with *R. groenlandicum* (16%), *C. calyculata* (8%), and *K. polifolia* (4%) higher in 2018. Also, *R. groenlandicum* abundances were different between years (*F* = 4.58, *p* = 0.038), but *C. calyculata* did not differ between the 2 years (*F* = 1.06, *p* = 0.308) (Figs. 2 and 3). Site differences are evident as seen in Kearl (along with JPH4) having more and Anzac less abundance of *R. groenlandicum* (Fig. 2), while Kearl had much less *C. calyculata* (Fig. 3). Although senior field personnel overlapped between the 2 years, plot assessments were carried out by different personnel over similar time periods (July 10–17, 2018; July 14–19, 2019).

#### Differences in species abundances between point-intercept and line transect assessment methods

**Fig. 4:**
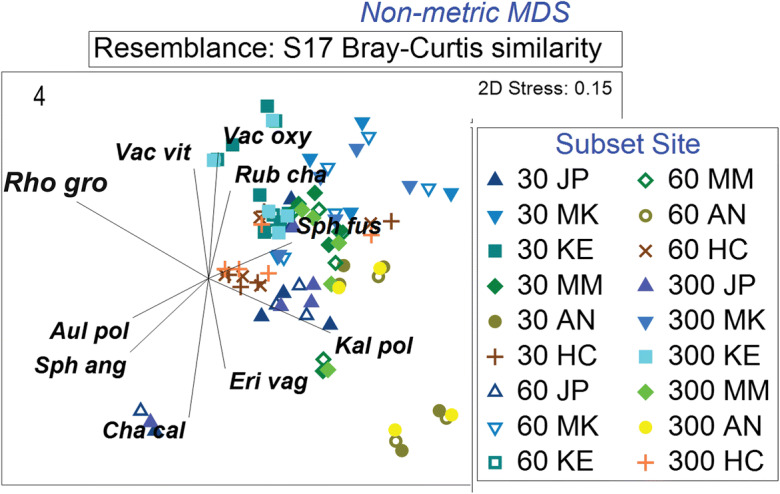
NMDS ordination of plant community composition for 2019 comparing similarities for data from the 30- and 60-point intercept analyses to the 300-cm line transects. Sites are designated by color and shape, and subsets are distinguished by color and color fill. Vectors describing species abundance patterns are shown as lines radiating from a common origin, offset to left of center of the ordination (Clarke & Gorley 2006). *AN* = Anzac, *HC* = Horse Creek, *JP* = JPH4, *KE* = Kearl, *MK* = McKay, *MM* = McMurray

Plot locations based on 30 and 60 points and those from data extracted from the 300-cm line transect for 2018 (not shown) and 2019 cluster close together on the NMDS ordination (Fig. 4). Similar to the line transect data, both the 30- and 60-point subsets revealed that the six bog sites were different from one another (*p* < 0.05). The subsets of 30 and 60 points also were not different from one another or the complete 300-point set (pseudo-*F* = 0.256, *p* = 0.985).

### Lichen chlorophyll and phaeophytin

**Fig. 5:**
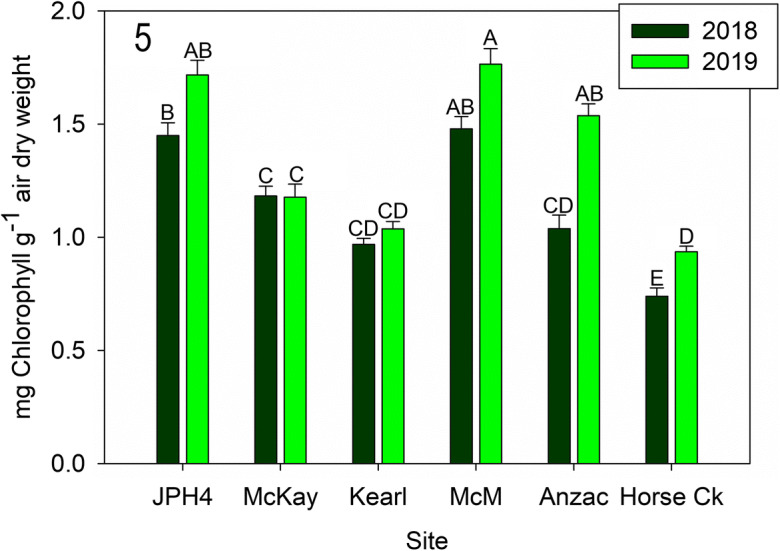
Mean (± S.E.) chlorophyll (chlorophyll *a* and *b*) for 2018 and 2019. Different letters indicate significantly different values (*p* < 0.05 Tukey’s test). *McM* = McMurray

Chlorophyll *a* and *b* concentrations for *Evernia mesomorpha* differed between sites (*F* = 79.240, *p* < 0.001), and year (*F* = 58.948, *p* < 0.001), with interactions between site and year (*F* = 7.701, *p* < 0.001). Overall, *E. mesomorpha* at Anzac (2019), JPH4, and McMurray had greater chlorophyll concentrations than at the remaining three sites. Chlorophyll concentrations were greater in 2019 than in 2018 at Anzac, and Horse Creek (Fig. 5).

**Fig. 6:**
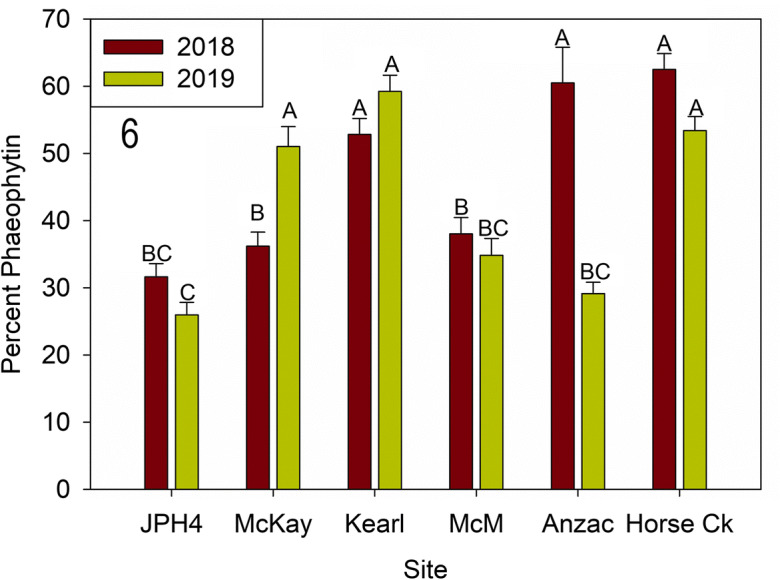
Mean (± S.E.) percent of phaeophytin *a* for 2018 and 2019. Different letters indicate significantly different values (*p* < 0.05 Tukey’s test). *McM* = McMurray

Percent phaeophytin *a* in *E. mesomorpha* also differed between sites (*F* = 42.750, *p* < 0.0001) and year (*F* = 10.151, *p* < 0.0001), with interactions between site and year (*F* = 15.172, *p* < 0.001) Although percent phaeophytin *a* was greater in 2018 than 2019 overall (Fig. 6), this difference was driven by one site (Anzac), with the remaining five sites either not different or the reverse (2019 greater than 2018 at McKay). When sites are compared, *E. mesomorpha* at Anzac (2018), Horse Creek, Kearl, and McKay (2019) had the highest percent phaeophytin contents.

**Fig. 7:**
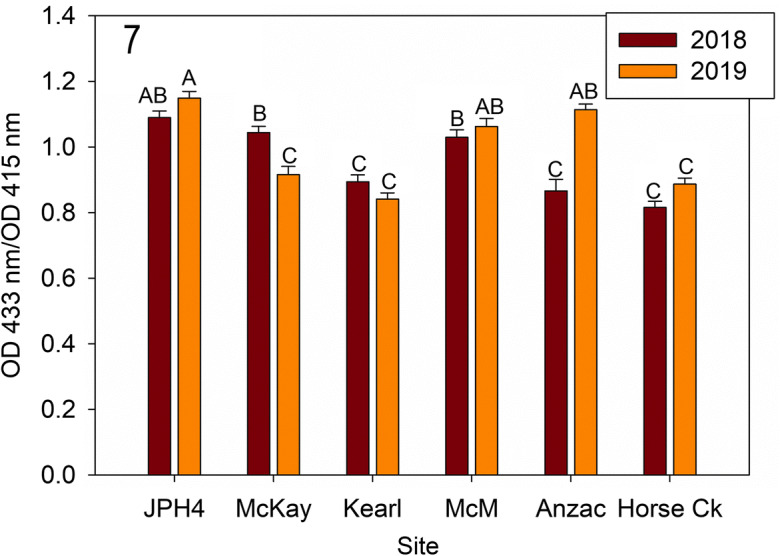
Mean (± S.E.) ratio of OD 433 to 415 nm (chlorophyll *a* to phaeophytin *a*) for 2018 and 2019. Different letters indicate significantly different values (< 0.05 Tukey’s test). *McM* = McMurray

The ratio of chlorophyll *a* to phaeophytin *a* in *E. mesomorpha* differed between sites (*F* = 43.283, *p* < 0.001), and year (*F* = 8.753, *p* = 0.003), with interactions between site and year (*F* = 16.250, *p* < 0.0001). High ratios were recorded at Anzac (2019), JPH4, McKay (2018), and McMurray (2019). Ratios between years were only different at Anzac (2019) and McKay (2019) (Fig. 7).

### Dominant shrub MPS and aboveground NPP

#### *Rhododendron groenlandicum* MPS and NPP

**Fig. 8:**
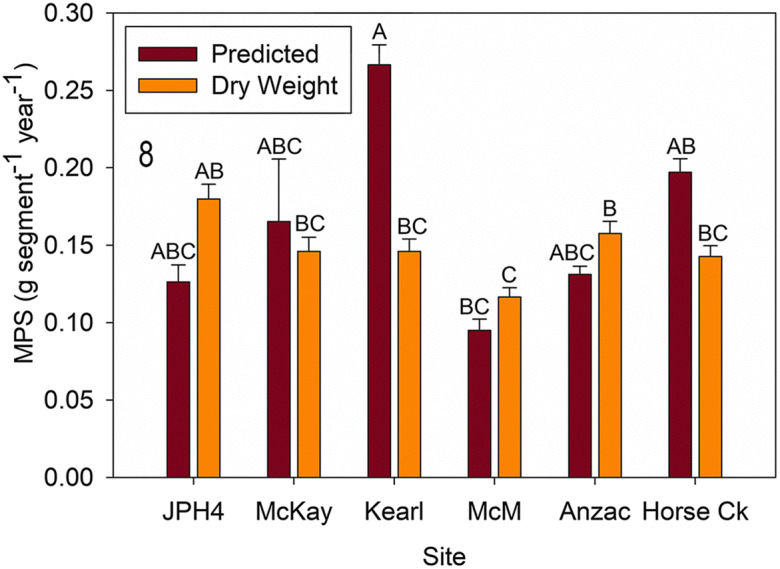
Mean (± S.E.) *Rhododendron groenlandicum* measured dry weight and predicted mass per new growth segment (MPS). Different letters indicate significantly different values (*p* < 0.05 Tukey’s test). *McM* = McMurray

Slopes for allometric growth equations modeled for each of the six sites were not different from one another or from the combined algorithm for 2019 (ANCOVA, *F* = 1.65, *p* = 0.158). A comparison between the predicted MPS (using the combined allometric equation) and actual dry weight (mass type) for *R. groenlandicum* (for 2019) revealed that site (*F* = 5.683, *p* < 0.0001) and the interaction between site and mass (*F* = 4.837, *p* = 0.0003) were significant, while mass type was not (*F* = 2.452, *p* = 0.118). Predicted MPS and actual dry weight were not different from one another except for Kearl (Fig. 8), where predicted MPS was 1.8 times higher than the actual dry weight (*p* < 0.001).

**Fig. 9:**
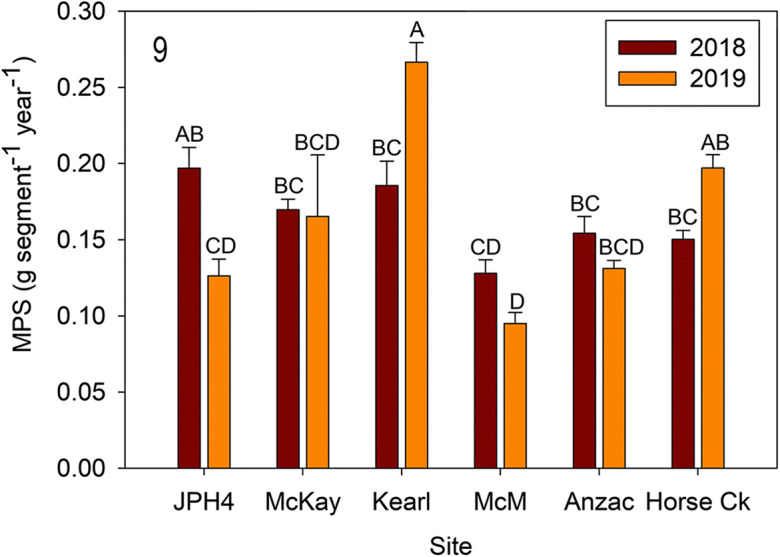
Mean (± S.E.) *Rhododendron groenlandicum* mass per new growth segment (MPS) for 2018 and 2019. Different letters indicate significantly different values (*p* < 0.05 Tukey’s test). *McM* = McMurray

Overall, *R. groenlandicum* predicted MPS was different between 2018 and 2019 (*F* = 4.467, *p* = 0.040), between sites (*F* = 10.961, *p* < 0.0001), and as interaction was detected between site and year (*F* = 16.220, *p* < 0.0001). Sites JPH4 and Kearl were different between 2018 and 2019 and MPS lower in 2019 than 2018 at JPH4 and higher in 2019 than 2018 at Kearl (Fig. 9).

**Fig. 10:**
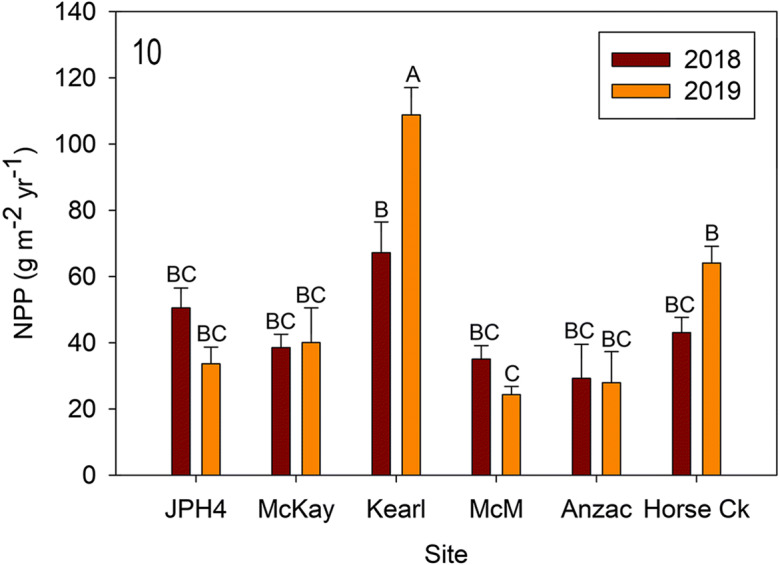
Mean (± S.E.) *Rhododendron groenlandicum* net primary production (NPP) for 2018 and 2019. Different letters indicate significantly different values (p < 0.05 Tukey’s test). *McM* = McMurray, Plant

Aboveground net primary production of *R. groenlandicum* varied from 25 to 68 g m^−2^ year^-1,^ with one outlier (Kearl 2019) at 115 g m^−2^ year^−1^ and differed between sites (*F* = 12.649, *p* < 0.0001) and between years (*F* = 7.022, *p* = 0.011), with an interaction between site and year (*F* = 17.722, *p* < 0.0001) (Fig. 10). Both site and year differences were driven by the high 2019 value at Kearl; otherwise, individual sites were not different, and only Kearl differed between years.

#### *Chamaedaphne calyculata* MPS and ANPP

**Fig. 11:**
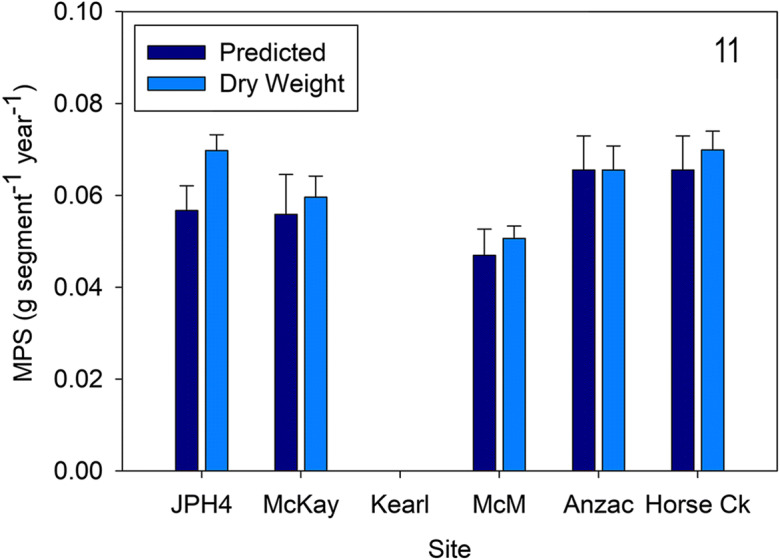
Mean (± S.E.) *Chamaedaphne calyculata* measured dry weight and predicted mass per new growth segment (MPS) for 2019. Different letters indicate significantly different values (*p* < 0.05 Tukey’s test). *McM* = McMurray

Similar to *Rhododendron groenlandicum*, slopes for modeled allometric growth equations (combined MPS vs. MPS for individual sites) for *C. calyculata* were not different for 2019 (ANCOVA, *F* = 0.91, *p* = 0.476). A comparison of modeled *C. calyculata* MPS and actual dry weight for 2019, site (*F* = 3.957, *p* = 0.009), mass type (*F* = 0.011, *p* = 0.917), and the interaction between site and mass (*F* = 0.204, *p* = 0.936) were not different. In particular, there were no differences between predicted MPS and dry weight for any of the five sites (*C. calyculata* absent at Kearl) (Fig. 11).

**Fig. 12:**
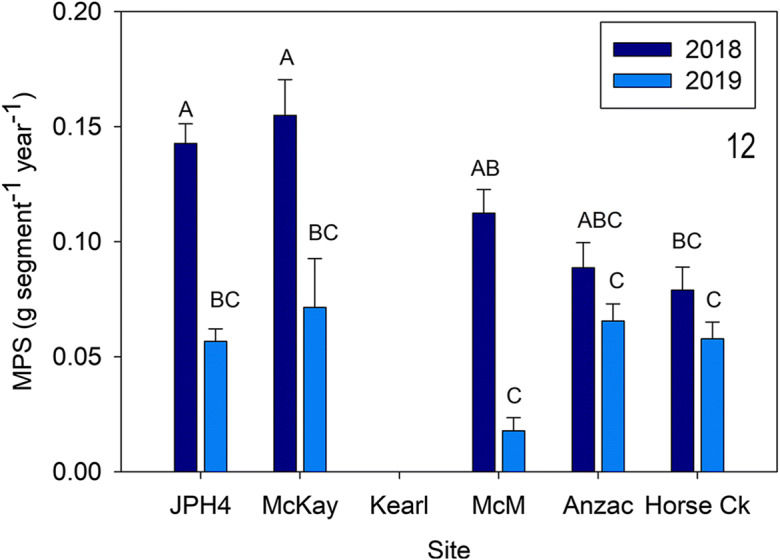
Mean (± S.E.) *Chamaedaphne calyculata* measured dry weight and predicted mass per new growth segment (MPS) for 2019. Different letters indicate significantly different values (*p* < 0.05 Tukey’s test). *McM* = McMurray

Overall, *C. calyculata* predicted MPS was greater in 2018 compared with 2019 (*F* = 65.654, *p* < 0.0001, also different were site (*F* = 3.957, *p* = 0.009),with an interaction between site and year (*F* = 3.885, *p* = 0.018). On average, *C. calyculata* MPS was two times higher in 2018 (mean = 0.116 g segment^−1^ year^−1^) than 2019 (mean = 0.058 g segment^−1^ year^−1^). Specifically, MPS was higher in 2018 than 2019 at JPH4, McKay, and McMurray (Fig. 12). In 2019, there were no differences between the five sites, whereas in 2018 only Horse Creek differed from JPH4 and McKay.

**Fig. 13:**
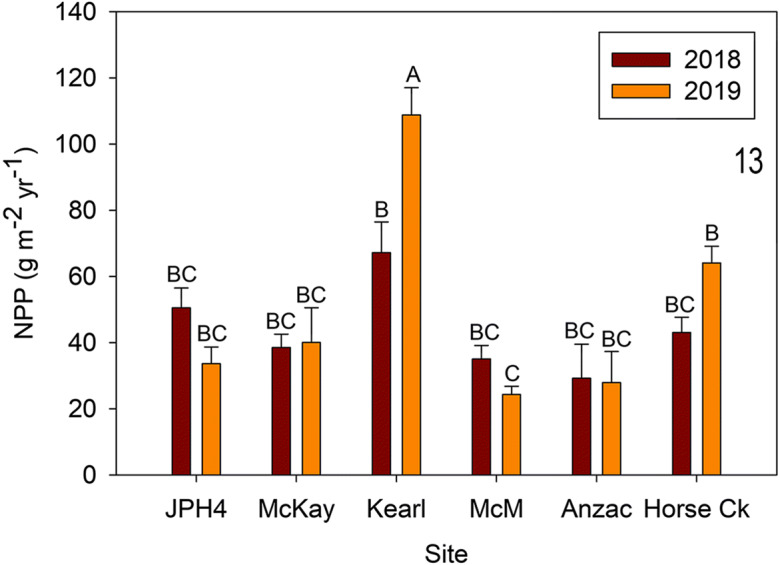
Mean (± S.E.) *Chamaedaphne calyculata* net primary production (NPP) for 2018 and 2019. Different letters indicate significantly different values (*p* < 0.05 Tukey’s test). *McM* = McMurray

*Chamaedaphne calyculata* NPP ranged from 3 to 24 g m^−2^ year^−1^. Both site (*F* = 2.932, *p* = 0.041) and year (*F* = 33.185, *p* < 0.0001) were different, and the interaction between site and year (*F* = 3.408, *p* = 0.024) was significant. As with MPS, NPP was 2.1 times higher—on average—in 2018 (mean = 15.73 g m^−2^ year^−1^) than 2019 (mean = 7.37 g m^−2^ year^−1^); however, among the five sites, only JPH4 and McMurray were different between years. Likewise, few differences were significant between sites, with only Horse Creek different from JPH4 (2018) (Fig. 13).

### *Sphagnum fuscum* growth and NPP

Plant density varied from 3.5 to 6.1 plants cm^−2^, with no difference between 2018 and 2019 (*p* = 0.367), differences between sites (*p* = 0.0003), and a nonsignificant year by site interaction (*p* = 0.134). Highest plant density was at Anzac, and lowest plant density was at JPH4 (Table 4). Stem mass density of *S. fuscum* ranged from 7.4 to 19.6 mg cm^−3^ and was higher in 2019 than in 2018 (*p* = 0.016), with differences between sites (*p* = 0.002), and a nonsignificant year by site interaction (*p* = 0.057) (Table 4). Stem mass density was higher at Anzac than at JPH4 or McKay (Table 5).

**Table 4:** *Sphagnum fuscum* plant density (# cm^−2^). Values are means ± standard errors (*n* = 5 for each year by site combination). Year or site means with the same letter do not differ significantly (ANOVA with a posteriori Tukey’s HSD mean comparisons, *p* = 0.05)

Site	2018	2019	Average across all years
JPH4	3.7 ± 0.2	3.1 ± 0.1	3.4 ± 0.1c
Kearl	3.5 ± 0.3	4.0 ± 0.3	3.8 ± 0.2bc
McKay	3.8 ± 0.5	3.9 ± 0.3	3.9 ± 0.3bc
McMurray	3.8 ± 0.3	3.9 ± 0.5	3.9 ± 0.2bc
Anzac	4.5 ± 0.3	6.5 ± 0.9	5.4 ± 0.5a
Horse Creek	5.1 ± 0.6	4.6 ± 0.5	4.8 ± 0.4ab
Average across all sites	4.1 ± 0.2a	4.3 ± 0.2a

**Table 5:** *Sphagnum fuscum* stem mass density (mg cm^−3^). Values are means ± standard errors (*n* = 5 for each year by site combination). Year or site means with the same letter do not differ significantly (ANOVA with a posteriori Tukey’s HSD mean comparisons, *p* = 0.05)

Site	2018	2019	Average across all years
JPH4	7.4 ± 0.6	9.5 ± 1.1	8.4 ± 0.7b
Kearl	7.6 ± 1.4	14.5 ± 1.5	11.0 ± 1.5ab
McKay	7.9 ± 1.5	9.3 ± 1.2	8.6 ± 0.9b
McMurray	11.5 ± 1.2	9.4 ± 1.1	12.8 ± 1.1ab
Anzac	11.5 ± 1.7	18.6 ± 0.7	14.6 ± 2.4a
Horse Creek	14.0 ± 3.1	13.7 ± 2.0	13.9 ± 1.7ab
Average across all sites	10.0 ± 0.8b	12.7 ± 1.0a	

Linear growth was more variable resulting in strong site differences. Linear growth ranged from 0.69 to 2.36 cm year^−1^and exhibited a significant year by site interaction (*p* < 0.0001). Linear growth was highest at JPH4 in 2018 and lowest at Horse Creek in 2018 (Table 6). Annual NPP of *S. fuscum* ranged from 93.8 to 380.3 g m^−2^ year^−1^ (Table 6), with a significant year by site interaction (*p* < 0.0001). At Kearl, Anzac, and Horse Creek, NPP was lower in 2018 than in 2019, with no difference between the 2 years at the other sites (Table 7).

**Table 6:** *Sphagnum fuscum* linear growth (cm year^−1^), assuming a 140-day growing season. Values are means ± standard errors (*n* = 5 for each year by site combination). Year or site means with the same letter do not differ significantly (nested ANOVA with a posteriori Tukey’s HSD mean comparisons, *p* = 0.05)

Site	2018	2019	Average across all years
JPH4	2.36 ± 0.21a	1.92 ± 0.49b	2.14 ± 0.14
Kearl	1.24 ± 0.30ef	1.08 ± 0.25f	1.16 ± 0.09
McKay	1.47 ± 0.42c-e	1.31 ± 0.20d–f	1.39 ± 0.10
McMurray	1.43 ± 0.12de	1.70 ± 0.19bc	1.56 ± 0.07
Anzac	1.51 ± 0.26cd	1.91 ± 0.18b	1.71 ± 0.09
Horse Creek	0.69 ± 0.14g	1.46 ± 0.23de	1.08 ± 0.14
Average across all sites	1.45 ± 0.10	1.56 ± 0.07

**Table 7:** *Sphagnum fuscum* NPP (g m^−2^ year^−1^), assuming a 140-day growing season. Values are means ± standard errors (*n* = 5 for each year by site combination). Year by site means with the same letter do not differ significantly (nested ANOVA with a posteriori Tukey’s HSD mean comparisons, *p* = 0.05)

Site	2018	2019	Average across all years
JPH4	173.3 ± 15.6c	182.2 ± 46.9bc	177.7 ± 10.5
Kearl	93.8 ± 22.7f	156.7 ± 36.0cd	125.2 ± 13.8
McKay	117.0 ± 33.2ef	132.4 ± 20.7de	124.7 ± 8.6
McMurray	164.0 ± 14.3c	159.5 ± 18.1c	161.8 ± 4.9
Anzac	173.2 ± 30.2c	375.8 ± 34.9a	274.5 ± 35.1
Horse Creek	96.8 ± 19.0f	200.3 ± 30.9b	148.5 ± 18.9
Average across all sites	136.3 ± 7.5	201.1 ± 16.9	

### *Picea mariana* leader length

*Picea mariana* leader lengths were significantly different between sites for the 1 year of measurement (*F* = 12.302, *p* < 0.001). *Picea mariana* individuals at Horse Creek had the shortest leader lengths compared with all other sites. The second shortest leader lengths were found at McKay, with no differences between McKay, Kearl, and McMurray. Although not significantly different from trees at Anzac, trees at JPH4 had the longest leader lengths. Leader lengths at Anzac, Kearl, and McMurray were not different from one another (Online resource 2).

### N and S deposition in 2018–2019

Over the past 20 years, gaseous emissions from oil sands operations have deposited NH_4_^+^, NO_3_^−^, and SO_4_^2^ on the surrounding landscape. Over this time period, N emissions have gradually increased, while since 2009 S emissions have declined to about half. Background deposition of N has been reported as 0.6 kg ha^−1^ year^−1^ and 0.4 kg ha^−1^ year^−1^ for sulfur (S) (Wieder et al. 2016a). During the 2-year time period related to this study (October 2017 to October 2019), N deposition averaged 1.31 kg ha^−1^ year^−1^ with NO_3_^−^-N 44% of the total. At our six sites annual deposition of NH_4_^+^-N varied from 0.52 to 0.96 kg ha^−1^ year^−1^, NO_3_^−^-N from 0.27 to 0.69 kg ha^−1^ year^−1^ with DIN varying from 0.82 to 1.51 kg ha^−1^ year^−1^. Sulfur deposition averaged 3.20 kg ha^−1^ year^−1^ and varied from 1.33 to 4.80 kg ha^−1^ year^−1^(unpublished data). Between Oct. 15, 2018, and May 15, 2019, 49% of the N deposition and 32% of the S deposition fell during the non-growing season.

### Site variability related to N and S deposition

**Fig. 14:**
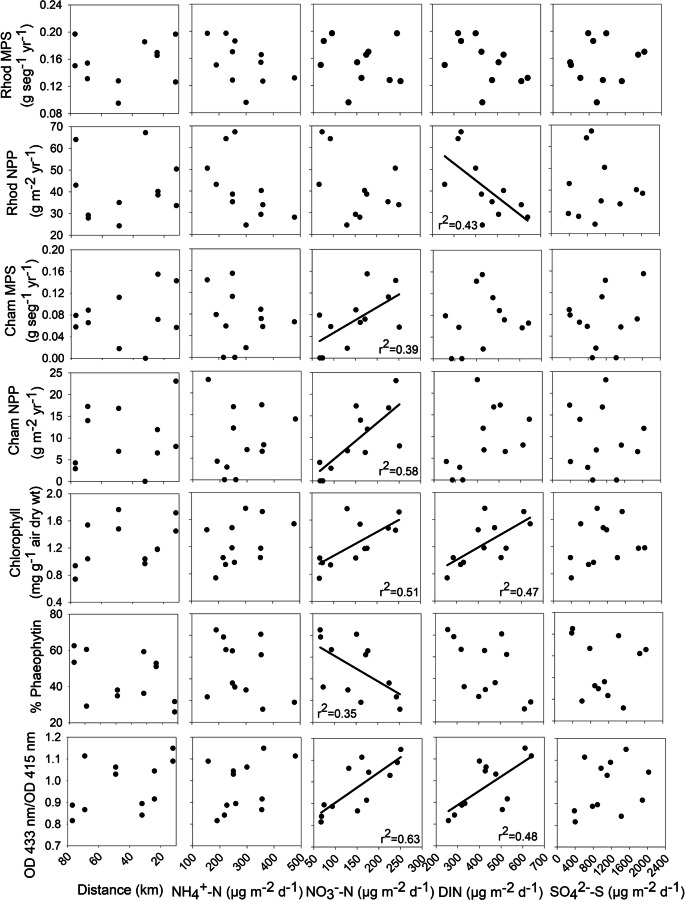
Plant/lichen responses for mass per segment (MPS) and net primary production (NPP) for *Rhododendron groenlandicum* (Rhodo) and *Chamaedaphne calyculata* (Cham), and chlorophyll, phaeophytin and the ratio of chlorophyll to phaeophytin for *Evernia mesomorpha* as a function of growing season N and S deposition for 2018 2019 at six research sites. Significant linear regressions shown

**Fig. 15:**
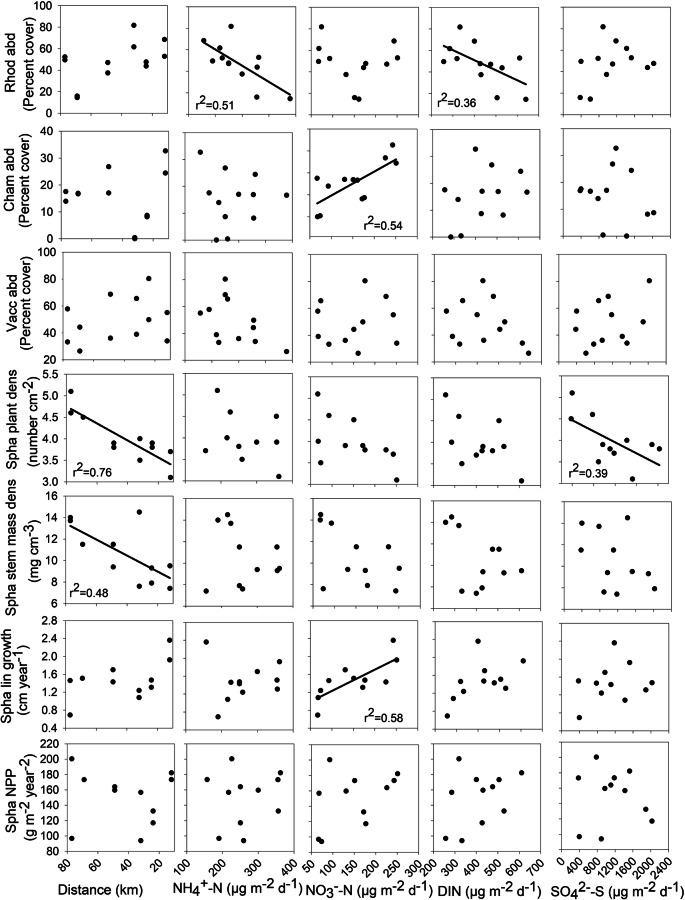
Plant/lichen responses for abundance (abd) for *Rhododendron groenlandicum* (Rhod), *Chamaedaphne calyculata* (Cham), and *Vaccinium oxycoccos* (Vacc), and plant density, stem mass density, linear growth, and NPP for *Sphagnum fuscum* (Spha) as a function of growing season N and S deposition for 2018 2019 at six research sites. Significant linear regressions shown

Among shrub attributes, *Rhododendron groenlandicum* abundance (*r*^2^ = 0.51) decreased with increases in NH_4_^+^-N and DIN deposition (*r*^2^ = 0.36; Fig. 15). Associated with these decreases in abundance was a decrease in NPP as DIN increased (*r*^2^ = 0.43); however, mass per segment showed no change (Fig. 14). *Chamaedaphne calyculata* abundance increased with an increase in NO_3_^−^-N deposition (*r*^2^ = 0.54) as well as mass per segment (*r*^2^ = 0.39) and NPP (*r*^2^ = 0.58; Fig. 13). *Evernia mesomorpha* chlorophyll concentration increased with increases in NO_3_^−^-N deposition (*r*^2^ = 0.51) and DIN (*r*^2^ = 0.47), along with a decrease in phaeophytin (*r*^2^ = 0.35) (and chlorophyll/phaeophytin ratio *r*^2^ = 0.63; Fig. 14). *Sphagnum* plant density (*r*^2^ = 0.76) and stem mass density (*r*^2^ = 0.48, the latter dependent of the former (cf. Wieder et al. 2016a, b) increased as distance from the upgrader stacks increased, while linear growth increased in association with increases in NO_3_^−^-N deposition (*r*^2^ = 0.58); however, *Sphagnum* NPP was not affected (Fig. 15). NO_3_^−^-N deposition was related to plant/lichen responses more than any other deposition-related factor (7 of the 14 significant responses), with six of the seven positive and the remaining negative phaeophytin one related to the positive chlorophyll increase in *Evernia* (Table 8).

**Table 8:** Results from linear regressions for fourteen plant/lichen responses as a function of five N and S deposition values from each of six research sites. *Rhododendron = R. groenlandicum; Chamaedaphne = C. calyculata; Vaccinium = V. oxycoccos;* chlorophyll, phaeophytin, and ratio of 433/425 for *Evernia mesomorpha; Sphagnum = S. fuscum.* Significant relationships (*p* < 0.05) in boldface

	Distance	NH_4_^+^-N	NO_3_^−^-N	DIN	SO_4_^2−^-S
*Rhododendron* MPS	*p* = 0.694; *r*^2^ = 0.018	*p* = 0.588; *r*^2^ = 0.034	*p* = 0.588; *r*^2^ = 0.034	*p* = 0.137; *r*^2^ = 0.228	*p* = 0.793; *r*^2^ = 0.008
*Rhododendron* NPP	*p* = 0.855; *r*^2^ = 0.039	*p* = 0.069; *r*^2^ = 0.321	*p* = 0.210; *r*^2^ = 0.169	*p = 0.028; r*^*2*^ *= 0.432*	*p* = 0.926; *r*^2^ = 0.001
*Chamaedaphne* MPS	*p* = 0.688; *r*^2^ = 0.017	*p* = 0.594; *r*^2^ = 0.029	*p* = 0.030; *r*^2^ = 0.389	*p* = 0.510; *r*^2^ = 0.045	*p* = 0.488; *r*^2^ = 0.049
*Chamaedaphne* NPP	*p* = 0.742; *r*^2^ = 0.011	*p* = 0.752; *r*^2^ = 0.010	*p = 0.004; r*^*2*^ *= 0.577*	*p* = 0.110; *r*^2^ = 0.235	*p* = 0.872; *r*^2^ = 0.003
Chlorophyll	*p* = 0.218; *r*^2^ = 0.148	*p* = 0.177; *r*^2^ = 0.174	*p = 0.010; r*^*2*^ *= 0.505*	*p = 0.014; r*^*2*^ *= 0.472*	*p* = 0.431; *r*^2^ = 0.063
Phaeophytin	*p* = 0.191; *r*^2^ = 0.165	*p* = 0.290; *r*^2^ = 0.111	*p = 0.044; r*^*2*^ *= 0.347*	*p* = 0.058; *r*^2^ = 0.314	*p* = 0.758; *r*^2^ = 0.010
433/415	*p* = 0.164; *r*^2^ = 0.184	*p* = 0.248; *r*^2^ = 0.131	*p = 0.002; r*^*2*^ *= 0.627*	*p = 0.013; r*^*2*^ *= 0.477*	*p* = 0.330; *r*^2^ = 0.095
*Rhododendron* abundance	*p* = 0.071; *r*^2^ = 0.290	*p = 0.010; r*^*2*^ *= 0.505*	*p* = 0.657; *r*^2^ = 0.020	*p = 0.040; r*^*2*^ *= 0.359*	*p* = 0.318; *r*^2^ = 0.010
*Chamaedaphne* abundance	*p* = 0.963; *r*^2^ = 0.000	*p* = 0.875; *r*^2^ = 0.003	*p = 0.007; r*^*2*^ *= 0.538*	*p* = 0.255; *r*^2^ = 0.127	*p* = 0.547; *r*^2^ = 0.037
*Vaccinium* abundance	*p* = 0.355; *r*^2^ = 0.086	*p* = 0.101; *r*^2^ = 0.247	*p* = 0.773; *r*^2^ = 0.009	*p* = 0.323; *r*^2^ = 0.098	*p* = 0.297; *r*^2^ = 0.108
*Sphagnum* plant density	*p = 0.001; r*^*2*^ *= 0.764*	*p* = 0.355; *r*^2^ = 0.095	*p* = 0.058; *r*^2^ = 0.343	*p* = 0.071; *r*^2^ = 0.317	*p = 0.039; r*^*2*^ *= 0.392*
*Sphagnum* bulk density	*p = 0.018; r*^*2*^ *= 0.478*	*p* = 0.564; *r*^2^ = 0.038	*p* = 0.095; *r*^2^ = 0.279	*p* = 0.157; *r*^2^ = 0.210	*p* = 0.176; *r*^2^ = 0.193
*Sphagnum* linear growth	*p* = 0.092; *r*^2^ = 0.284	*p* = 0.815; *r*^2^ = 0.006	*p = 0.006; r*^*2*^ *= 0.584*	*p* = 0.092; *r*^2^ = 0.283	*p* = 0.486; *r*^2^ = 0.055
*Sphagnum* NPP	*p* = 0.883; *r*^2^ = 0.003	*p* = 0.653; *r*^2^ = 0.023	*p* = 0.199; *r*^2^ = 0.177	*p* = 0.276; *r*^2^ = 0.130	*p* = 0.860; *r*^2^ = 0.004

## Discussion

Both plant community structure and function are attributes of equal importance in assessing the health of ecosystems. Plant community structure can be examined through assessment of plant species abundances that can serve as indicators of environmental changes occurring at the site level. Functional attributes that include growth and net primary production also are means of assessing how environmental changes affect the overall health of the ecosystem long before changes are evident in tissue concentrations. Accurate monitoring protocols are essential for these assessments as are baseline data that reflect natural annual variation.

In N-limited habitats increasing N deposition could lead to, first, stimulation of growth and increased NPP, and secondly, when N availability exceeds demands of growth, to increases in tissue N-concentrations. Over the past several decades, considerable research has examined either growth or tissue concentration responses to experimentally applied N and S, including studies in Europe (reviewed by Bobbink and Hettelingh 2011) and eastern North America (e.g., Bubier et al. 2011; Juutinen et al. 2015). Nearly all of these studies were done in regions having historically high N deposition and in bogs that have quite different flora than in Alberta, making comparisons difficult. Bogs in continental North America (west of central Ontario to the Canadian Rocky Mountains) are unique in being dominated by the tree species, *Picea mariana*, and the tall shrub, *Rhododendron groenlandicum*, both species endemic to North America. Whereas western boreal bogs have an open canopy of *P. mariana* (Wieder et al. 2009), bogs of eastern North America either have no trees or only small scattered individuals of the species (Glaser and Janssens 1986). Bogs of Eurasia are either treeless or have scattered individuals of *Pinus sylvestris* and hummocks of *Calluna vulgaris* (Ruuhijärvi 1983; Sjörs 1983), both species that do not occur naturally in North America. These floristic differences may be important to recognize in any long-term evaluation of plant responses to N deposition.

Our objective was to develop a protocol that can be used to quantify important attributes of bog structure and function. Recent experimental studies by Wieder et al. (2019) explored how a bog ecosystem was affected by increasing levels of N deposition, and that study has provided the framework from which we chose appropriate attributes to include in a monitoring protocol. We tested our protocol by examining plant community structure using a line transect and compared the results to a more limited point transect method. We developed a non-destructive field-based method for assessing plant annual growth and production utilizing the dominant shrubs present at each site. We used an epiphytic lichen species to develop a suitable technique for using lichen chlorophyll/phaeophytin ratios as an indicator of lichen health, and examined the variability in *Sphagnum fuscum* growth and NPP. We used leader length of *Picea mariana* to examine variability in tree growth.

Plant community composition (Figs. 1, 2, 3, and 4): Ombrotrophic bogs are among the most species poor plant communities in the boreal region (Vitt 2006). Even though we recorded only 22 species, our six sites differed in individual species abundances. The sites differed largely owing to variability in shrub abundances, with high consistency of individual plots with each site Over the 2 years of measurement, the six sites differed between years, with shrub cover greater (by 1.23 times) in 2018 compared with 2019. Average daily temperatures in 2018 were approximately 10% higher than in 2019 for the growing season up to July 1 (data from weather station at the Anzac site, not shown). The significant plant community change between years was related to lower, but variable vascular plant cover in 2019, varying from 17 to 34% lower overall cover. Especially noteworthy was the large decrease in *Vaccinium oxycoccos* frequency (51% lower in 2019). Other species also contributed to the decrease in abundance ranging from 4 to 16% lower; however, these lower abundances in 2018 were not consistent between sites. For example, the dominant species, *Rhododendron groenlandicum*, was higher at two sites, but only slightly higher at three sites and slightly lower at one site; however, the overall effect of this variation was a 17% increase in *R. groenlandicum* abundance when all sites are considered together. We determined that our 2-year line transect data were sufficiently sensitive to show that bog communities vary, both between sites and over time. We suggest that a line transect method has appropriate sensitivity for assessing changes in bog plant communities, but that site variability must be taken into account in future monitoring by assessing changes at individual sites over a temporal frame.

Data collection in the field for line transects is time-consuming and rather tedious. We analyzed our transect data to determine if we could reduce the effort in the field by reducing the line transect data to points along the line transect. Surprisingly, point observation methods yielded no differences from the line transect and provide ordinations that position plots from point and line transects in close proximity to one another for both 2018 and 2019 (Fig. 4). We suggest that the similarity of these methods indicate that at the hummock-scale in bogs, individual species occurrences are quite consistent along the hummock surface and that when field effort is a concern, the point-intercept method may provide sufficient data.

Lichen chlorophyll and phaeophytin (Figs. 5, 6, and 7): Chlorophyll *a/b* concentrations were variable, differing some two-fold (ca. 0.7 to 1.7 mg g^−1^ chlorophyll) and in general, formed two groups: Anzac-2019, JPH4, and McMurray in one group with relatively high chlorophyll concentrations, while Anzac-2018, Horse Creek, Kearl, and McKay had low concentrations. Three of the six sites had significant differences between years. Likewise, phaeophytin varied from ca. 30% to over 60% between sites with two mostly similar groups and year differences as those of chlorophyll *a/b* concentrations. Groups and years with high chlorophyll content had low phaeophytin concentrations. The ratios of chlorophyll to phaeophytin were less variable, but overall showed the same patterns.

*Rhododendron* growth and ANPP (Figs. 8, 9, and 10): Actual mass per new growth segment (MPS—as a surrogate for new growth) was measured destructively in 2019 and varied from 0.12 to 0.18 g segment^−1^ for the six sites. Two sites with high MPS (Anzac [0.16] and JPH4 [0.18]) differed from McMurray (0.12) with a low value. We produced algorithms for MPS for the six individual sites and an algorithm for all sites combined. Slopes for individual site algorithms were not different values from the overall one; thus, one generalized algorithm appears to be sufficient for all of our sites (*r*^2^ = 0.85), and probably useful for all bog sites in the region.

Except for Kearl, predicted 2019 MPS values were not different from actual values. Comparing the 2 years, predicted MPS revealed only JPH4 (lower in 2019) and Kearl (higher in 2019) had differing MPS values. The difference at JPH4 between 2018 and 2019 seems real as the variation is within that from other sites and although not significant, the abundance of *Rhododendron* at this site was also lower (Fig. 2). However, the large difference at Kearl is seemingly anomalous, as the abundance values were the reverse and the MPS for 2019 was much outside of the ranges of all the remaining sites. We attribute this high value to destructively sampled *Rhododendron* leaves and stems being smaller than those from plants within the quadrats. Our MPS values (0.12 to 0.18 g segment^−1^) are somewhat higher than those reported by Wieder et al. (2019) (ca. 0.06–0.12) for Mariana Bog, a nearby site with less abundance of *Rhododendron*.

Comparing our six bog sites to the Mariana bog site used in Wieder et al. (2019) revealed differences in stem densities for *Rhododendron*. Stem densities varied from 174 (Anzac) to 355 (Kearl) stems m^−2^ in 2018 and from 205 (Anzac) to 409 (Kearl) stems m^−2^ in 2019. Five of the six sites had stem counts from 1 to 54 (Kearl) stems greater in 2019, with one site (McMurray slightly lower (− 16), but overall stem counts did not differ in the 2 years. These stem densities are higher compared with those reported from (Wieder et al. 2019) with stem densities around 50 stems m^−2^. The lower stem densities reported in Wieder et al. (2019) yielded much lower NPP (ca. 2–15 g m^−2^ year^−1^). Actual NPP from our six sites varied from 25 g m^−2^ year^−1^ (McMurray 2019) to 70 g m^−2^ year^−1^ (Kearl 2018) and 110 g m^−2^ year^−1^ (Kearl 2019). Both MPS and stem density are key components of NPP for *Rhododendron*. Our predicted MPS plus stem density count predicted wide variation in NPP between sites, but little variation between years (only the anomalous Kearl 2019 reading was different between years), suggesting that like our line transect analysis, comparisons over time must be addressed for individual sites.

*Chamaedaphne* growth and ANPP (Figs. 11, 12, and 13). We used similar methods for MPS and NPP of *C. calyculata*, the shrub with moderate abundances at several of the sites (absent at Kearl). Our algorithm results are similar to those of *Rhododendron* (*r*^2^ = 0.91) with an overall algorithm sufficient to predict MPS for *Chamaedaphne* (our *r*^2^ values are identical to those for these two species in Wieder et al. (2019)). Predicted mass growth in 2019 values was not different from actual measured ones and varied from 0.05 to 0.07 g segment^−1^ for the five sites. Predicted values for 2018 were higher at three of the five sites (JPH4, McKay, and McMurray) compared with those from 2019. NPP for *Chamaedaphne* were much lower than those for *Rhododendron*, varying from 2.5 (Horse Creek 2019) to 23 g m^−2^ year^−1^ (JPH4 2018) and are comparable to those from Mariana Lake Bog (4 to 16 g m^−2^ year^−1^; Wieder et al. 2019).

*Sphagnum* NPP (Tables 4, 5, 6, and 7): The ground layer of bogs is of fundamental importance to ecosystem function and the ground layer of mature bogs is dominated by species of *Sphagnum* (Belland and Vitt 1995). Large expansive hummocks have a continuous layer of *S. fuscum*, while wetter hollows can have areas dominated by *S. angustifolium* and *S. magellanicum*. In boreal Canada, a recent study demonstrated that while linear growth was not affected by increased N deposition, plant density and stem mass density were resulting in a decrease in NPP (Wieder et al. 2019). That study reported at Mariana Bog the NPP for control sites was 281 g m^−2^ year^−1^, and linear growth 2.3 cm year^−1^ over the course of the 5-year study. Our study reported *Sphagnum* NPP that averaged 136.3 in 2018 and 201.0 in 2019 g m^−2^ year^−1^ and ranged from 94 to 376 g m^−2^ year^−1^, while linear growth averaged 1.45 in 2018 and 1.56 in 2019 cm year^−1^ and ranged from 0.69 to 2.36 cm year^−1^. Compared with the Mariana Bog, *Sphagnum* linear growth and NPP for our sites were lower. Thus, our sites had greater shrub abundances and shrub production and less ground layer growth and production. *Sphagnum* performance was more variable than that of *Rhododendron* and *Chamaedaphne*, with high variability among the sites in both linear growth and NPP. In general, NPP at the six sites separated into two groups in 2018 and into three groups in 2019; four of the sites were different between the 2 years, again indicating that monitoring must compare individual sites over time.

We examined leader lengths of *Picea mariana* by measuring the previous year’s leader. Although sites were different, this difference was driven by two sites (Anzac and JPH4) having long leaders (> 4 cm) vs. Horse Creek having short ones (< 2 cm). We are unable to make a recommendation based on these limited data, and measurements in additional years are necessary.

The significant site variation in all of the plant and lichen attributes led us to consider whether some of this variation is related to differences in either N or S deposition. Background deposition for the oil sands region has been reported as 0.6 kg ha^−1^ year^−1^for N and 0.4 kg ha^−1^ year^−1^ for S, with our six sites varying from 0.82– to 1.51 kg ha^−1^ year^−1^ for N and 1.33 to 4.80 kg ha^−1^ year^−1^for S. These increases in N, although 1.4 to 2.5 fold higher than regional background levels remain relatively low given that the critical load recommended by Wieder et al. (2019) of 3 N kg ha^−1^ year^−1^ and that of Pardo et al. (2011) of 4–6 N kg ha^−1^ year^−1^ for epiphytic lichens of the Northern Forest Ecoregion. Significant decreases in *Rhododendron* abundance and NPP and increases in *Chamaedaphne* MPS and NPP provide evidence that the shrub component is sensitive to changes in N deposition. Likewise, increases in *Evernia* chlorophyll concentration coupled to a decrease in phaeophytin concentration are early indicators of depositional changes. Concentrations of chlorophyll and phaeophytin, along with their ratios, have been used extensively in the literature as indicators of physiological health of lichen thalli (Ronen and Galun 1984). Increased chlorophyll concentrations are an indication of an early fertilization response to increased N exposure, with increased chlorophyll allowing for increased photosynthesis and increased growth (Ra et al. 2005) and may be an early warning response to increased N deposition (Berryman et al. 2016; Johansson et al. 2010). However, long-term chronic exposure to atmospheric stressors such as N and S may lead to increased phaeophytin, an increase in tissue concentrations, and eventual death of the lichen thalli.

Responses to increases in S appear to be limited with only *Sphagnum* plant density affected; however, this response is strongly related to distance from the upgrader stacks. The decrease in *Sphagnum* plant density was coupled to an increase in linear growth resulting in no change in NPP. Based on these 2 years of data, of the 14 plant/lichen responses we monitored, variation in nine was significantly related to N deposition, only one related to S deposition, and two related to distance from the upgrader stacks (and one of these associated with S deposition). We found that between 35 and 76% of the variability present in these nine responses could be explained by N or S deposition.

## Conclusion

Considerable research conducted across Europe has provided widespread agreement that increasing N deposition has negative consequences for bog structure and function (reviewed in Wieder et al. 2019), and these responses have been examined in North America (Bubier et al. 2007, 2011; Rochefort et al. 1990). In general, most important of these responses were changes in the ground layer and vascular plants. Especially noteworthy was the increase in abundance of vascular plants and decrease or change in the species of *Sphagnum* at higher N concentrations. Over the 2 years of our study we have assessed the responses of two shrubs, the epiphytic lichen *Evernia mesomorpha* and *Sphagnum fuscum*, to assess the responses in six boreal bogs in order to develop a protocol for monitoring future effects of changing atmospheric N and S deposition regimes. The ombrogenous condition of bog ecosystems provides a unique opportunity for these assessments; however, at present no structured protocol has been available. We conclude the following: (1) Bog communities are species poor with high degree of similarity for vascular plant occurrences; however, differences in bryophyte species and variation in vascular plant abundances (especially that of shrubs) indicate that bog sites are individually distinct, and monitoring over a temporal framework must take this site variation into account. (2) At the plot level, both line-intercept and point-intercept methods of assessing plant abundances provided similar results, with almost all plots within a site having high similarity and indicating that individual sites have highly uniform structure. (3) Sites also are variable between years especially in shrub abundances that couple to differences in annual growth and ANPP. Also, variable between years and sites is *Sphagnum* NPP. (4) We developed allometric growth equations for non-destructively predicting annual MPS and ANNP for two dominant shrubs. These equations predicted differences in annual NPP between years (higher in 2018 compared with 2019) and between many of the sites. (5) We found the lichen *Evernia mesomorpha* was abundant at all of the sites and easy to collect in the field. Laboratory analyses of chlorophyll and phaeophytin contents were straight-forward and yielded data that indicate clear differences between years and sites. (6) *Picea mariana* leader lengths are variable among the sites, but we found it difficult to find suitable trees and to be sure that we measured the correct bud scars; thus, we have less confidence in this measure. Based on these results we recommend a monitoring protocol of (a) sites with 5 permanent plots each with (b) 30 or 60 point-intercepts along a 300-cm line transect followed by multivariate analysis, (c) two 1-m^2^ plots non-destructively sampled annually for *Rhododendron groenlandicum* and *Chamaedaphne calyculata* annual growth, (d) *Evernia mesomorpha* sampled for chlorophyll/phaeophytin analysis, and (e) NPP of *Sphagnum fuscum* determined from cranked wires. Additionally, variation in many responses at the site level are correlated to patterns of N deposition in the oil sands area, suggesting that attributes of shrubs, the lichen *Evernia mesomorpha* and *Sphagnum fuscum*, are useful indicators of changes in N, and to a lesser extent, S deposition.

## Electronic supplementary material

ESM 1 (PDF 2181 kb)

ESM 2 (PDF 119 kb)
