# Supplementary material for: A protocol for monitoring plant responses to changing nitrogen deposition regimes in Alberta bogs
Source: Environ Monit Assess. 2020 Nov 2;192(11):743. doi: 10.1007/s10661-020-08645-z (PMC7606289; doi:10.1007/s10661-020-08645-z)
Supplement: Supplementary file 1 — (PDF 2181 kb) [file 10661_2020_8645_MOESM1_ESM.pdf]

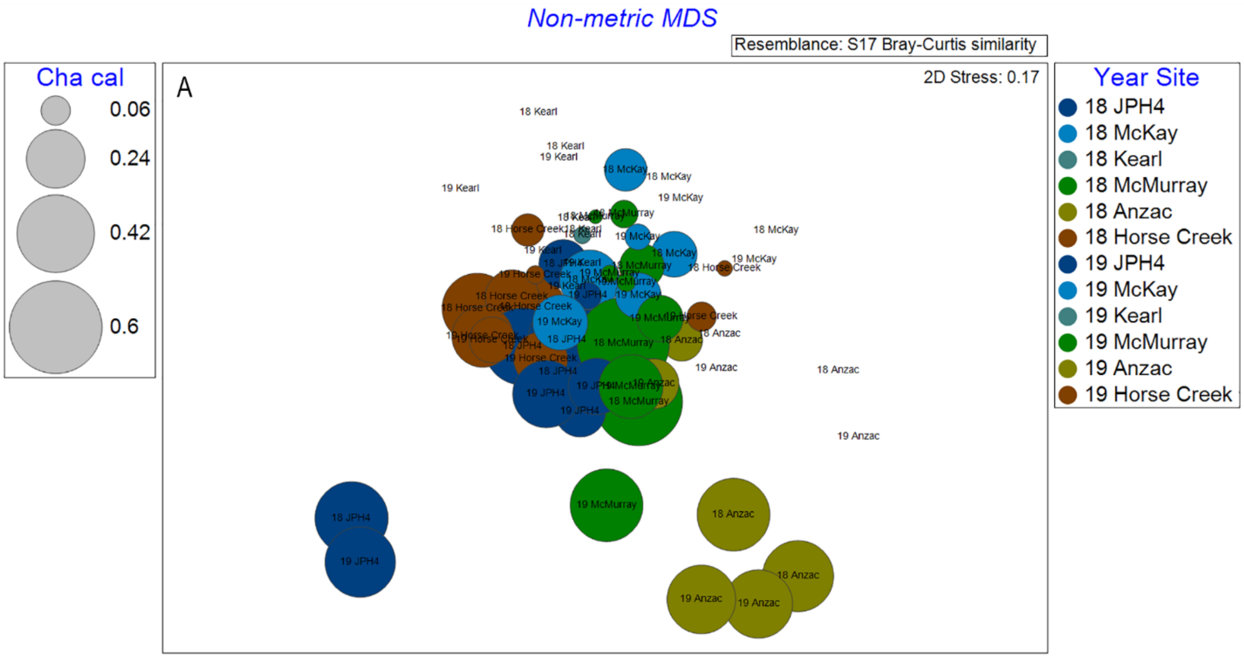
Non-metric MDS
Resemblance: S17 Bray-Curtis similarity
Cha cal
0.06
0.24
0.42
0.6
A
2D Stress: 0.17
Year Site
18 JPH4
18 McKay
18 Kearl
18 McMurray
18 Anzac
18 Horse Creek
19 JPH4
19 McKay
19 Kearl
19 McMurray
19 Anzac
19 Horse Creek

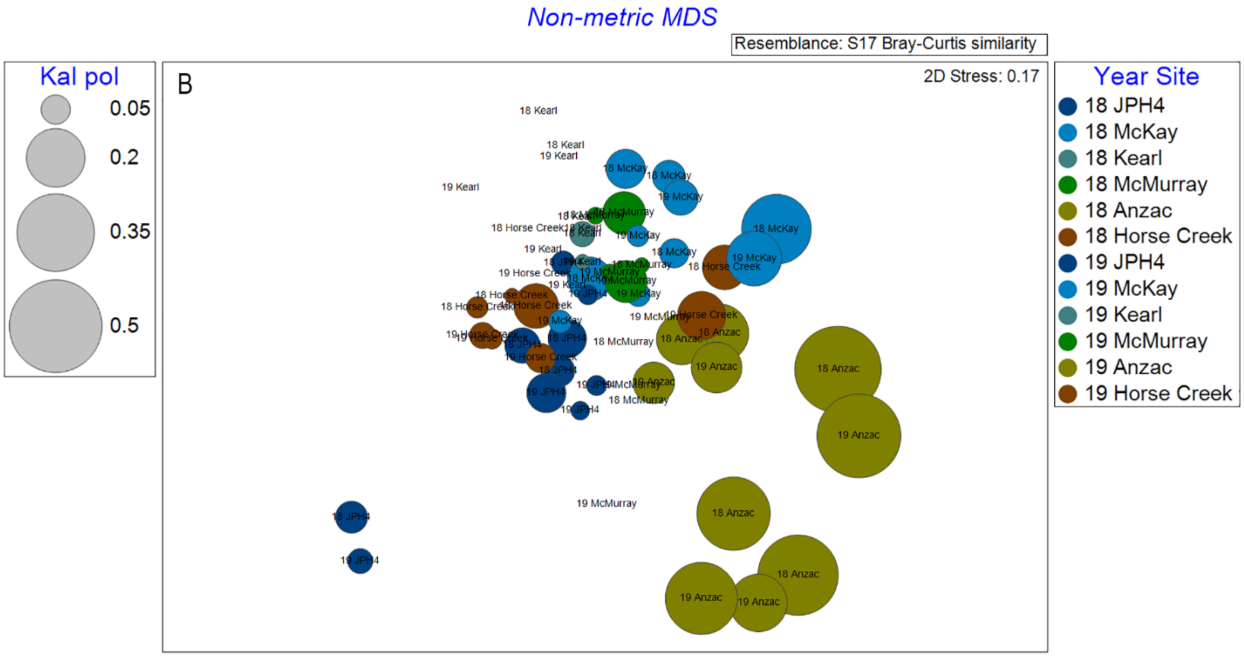
Non-metric MDS
Resemblance: S17 Bray-Curtis similarity
Kal pol
0.05
0.2
0.35
0.5
B
2D Stress: 0.17
Year Site
18 JPH4
18 McKay
18 Kearl
18 McMurray
18 Anzac
18 Horse Creek
19 JPH4
19 McKay
19 Kearl
19 McMurray
19 Anzac
19 Horse Creek

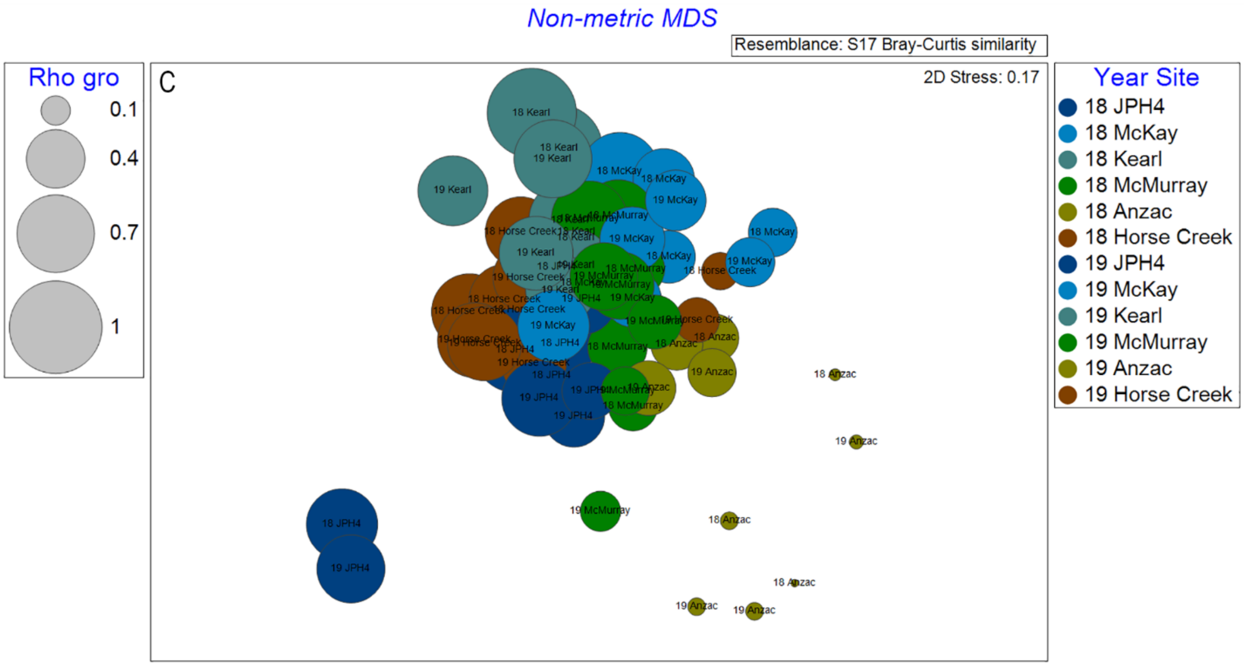
Non-metric MDS
Resemblance: S17 Bray-Curtis similarity
C
2D Stress: 0.17
Rho gro
0.1
0.4
0.7
1
Year Site
18 JPH4
18 McKay
18 Kearl
18 McMurray
18 Anzac
18 Horse Creek
19 JPH4
19 McKay
19 Kearl
19 McMurray
19 Anzac
19 Horse Creek

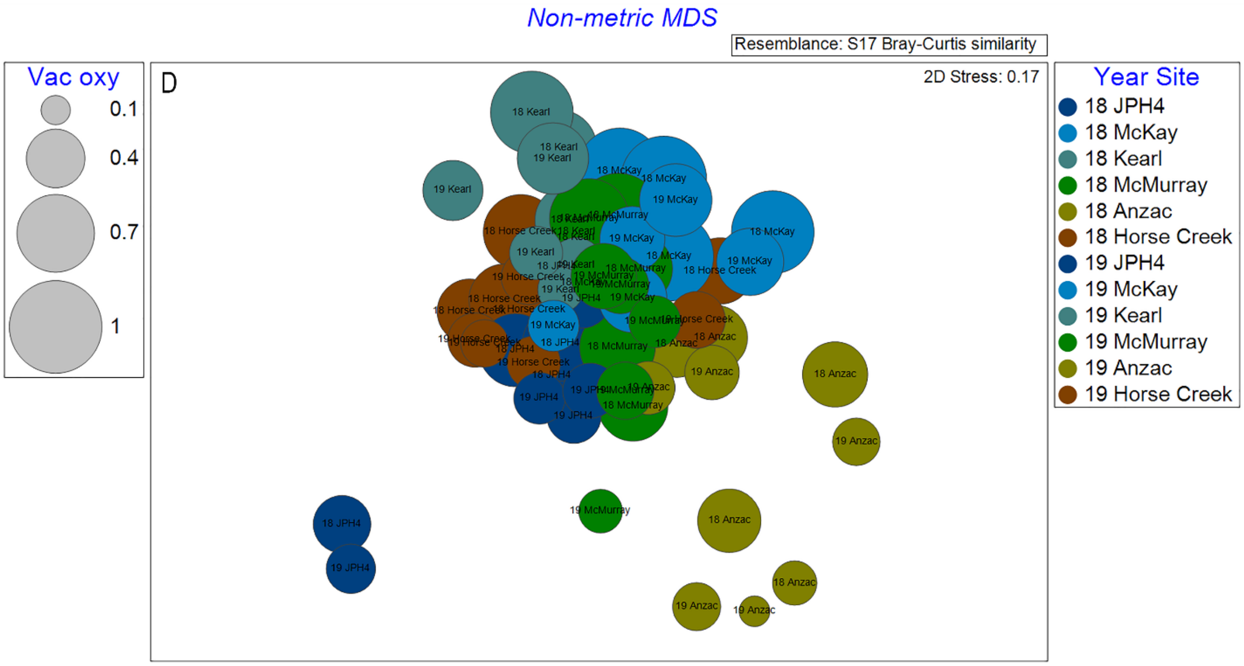
Non-metric MDS
Resemblance: S17 Bray-Curtis similarity
D
2D Stress: 0.17
Vac oxy
0.1
0.4
0.7
1
Year Site
18 JPH4
18 McKay
18 Kearl
18 McMurray
18 Anzac
18 Horse Creek
19 JPH4
19 McKay
19 Kearl
19 McMurray
19 Anzac
19 Horse Creek

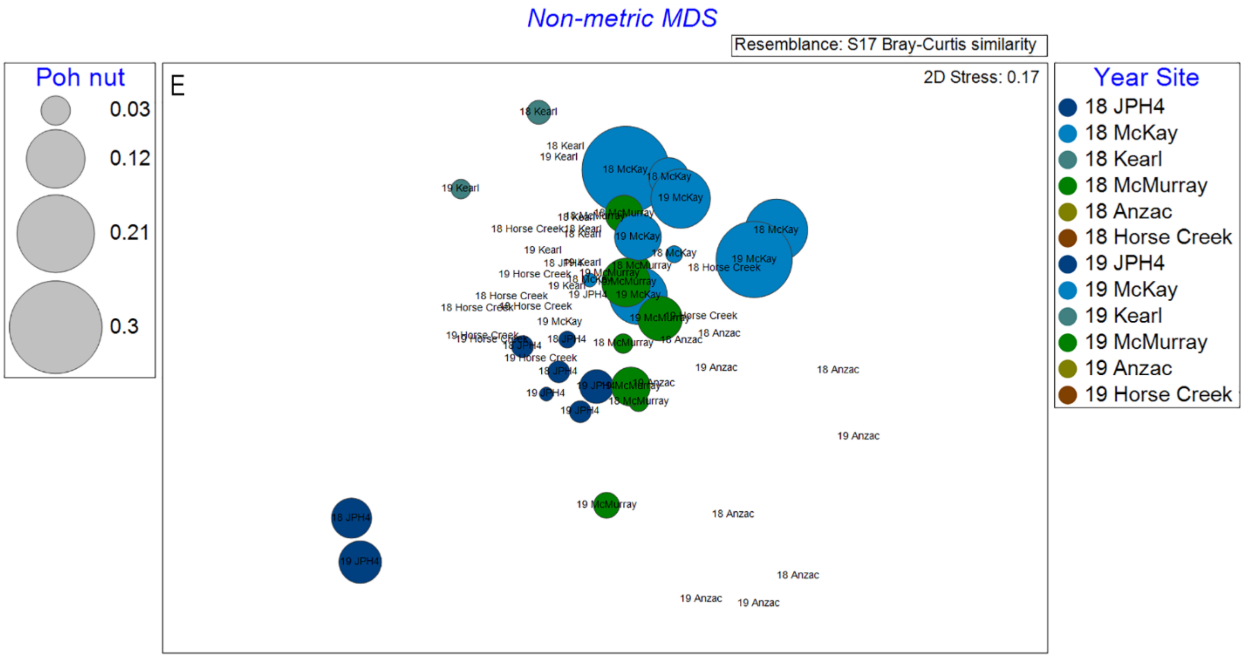

Non-metric MDS
Resemblance: S17 Bray-Curtis similarity
E
2D Stress: 0.17
Poh nut
0.03
0.12
0.21
0.3
Year Site
18 JPH4
18 McKay
18 Kearl
18 McMurray
18 Anzac
18 Horse Creek
19 JPH4
19 McKay
19 Kearl
19 McMurray
19 Anzac
19 Horse Creek

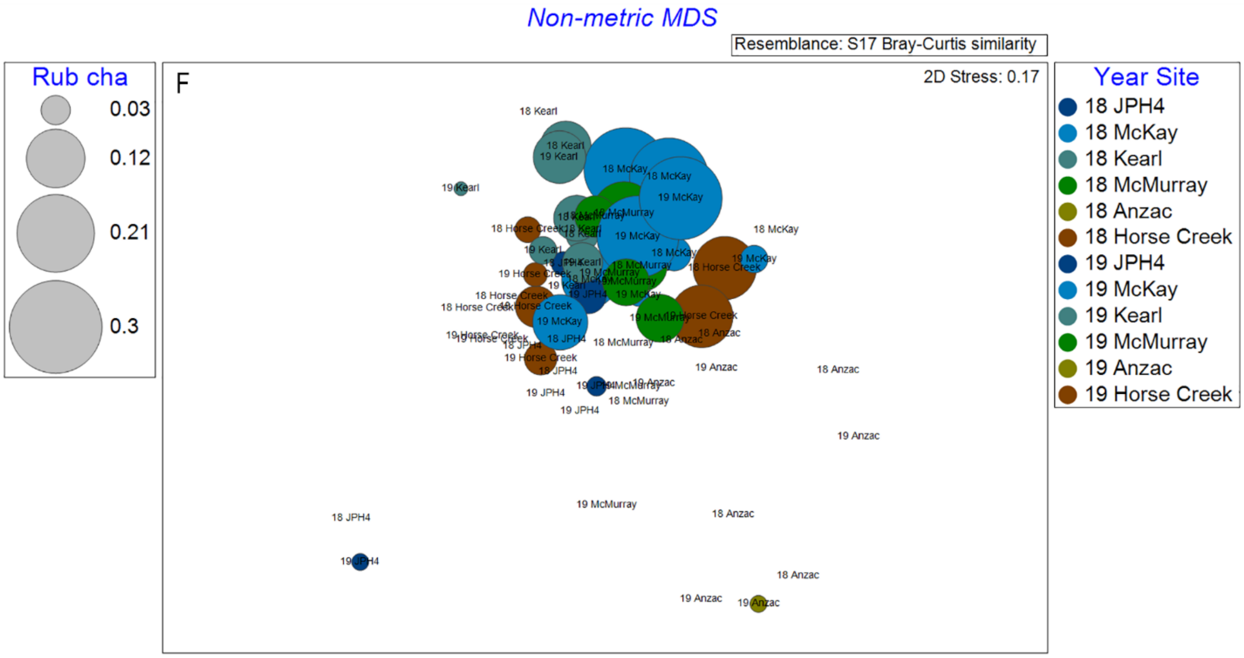

Non-metric MDS
Resemblance: S17 Bray-Curtis similarity
F
2D Stress: 0.17
Rub cha
0.03
0.12
0.21
0.3
Year Site
18 JPH4
18 McKay
18 Kearl
18 McMurray
18 Anzac
18 Horse Creek
19 JPH4
19 McKay
19 Kearl
19 McMurray
19 Anzac
19 Horse Creek

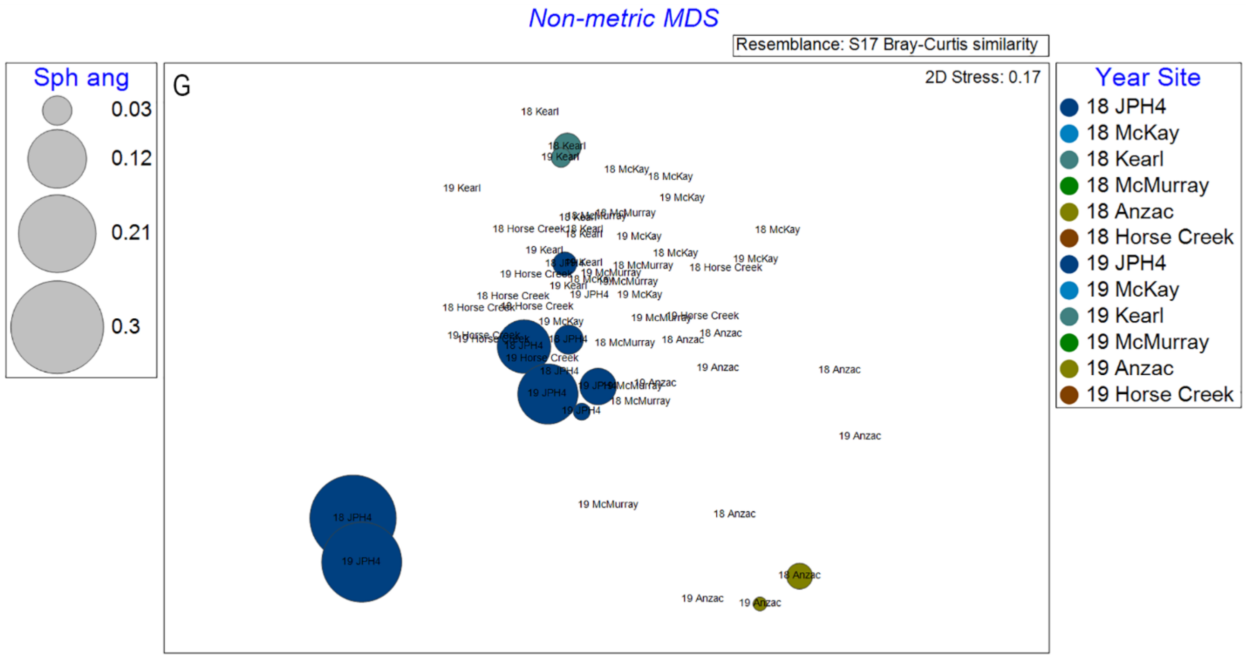

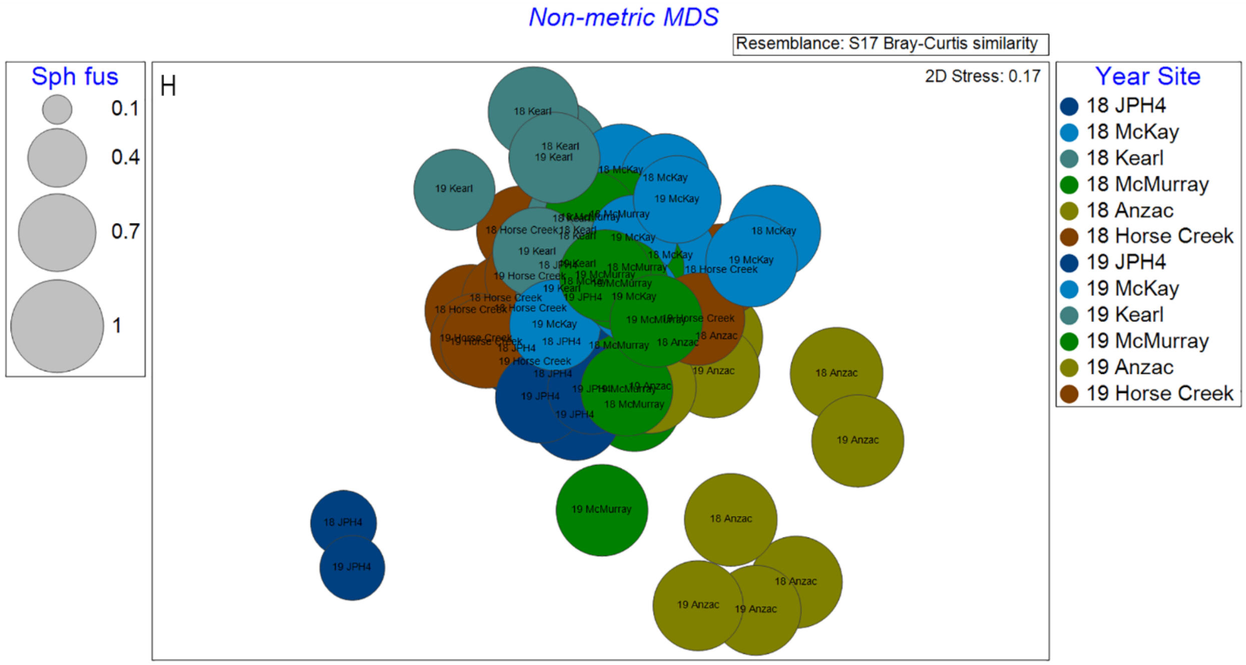

Supplementary Figure S1A-H. .Differences in dominant species at the six bog sites plotted on the NMDS ordination. Size of the bubble indicates abundance, color indicates site. Fig. A. *Chamaedaphne calyculata*. Fig. B. *Kalmia polifolia*. Fig. C. *Rhododendron groenlandicum*. Fig.D *Vaccinium oxycoccos*. Fig. E. *Pohlia nutans*. Fig. F. *Rubus chamaemorus*. Fig. G. *Sphagnum angustifolium* Fig. H. *Sphagnum fuscum*

A protocol for monitoring plant responses to changing nitrogen deposition regimes in Alberta bogs;

Monitoring and Assessment, Dale H. Vitt (dvitt@siu.edu), Melissa House, Samantha Kitchen, R. Kelman Wieder
