# Supplementary material for: A protocol for monitoring plant responses to changing nitrogen deposition regimes in Alberta bogs
Source: Environ Monit Assess. 2020 Nov 2;192(11):743. doi: 10.1007/s10661-020-08645-z (PMC7606289; doi:10.1007/s10661-020-08645-z)
Supplement: Supplementary file 2 — (PDF 119 kb) [file 10661_2020_8645_MOESM2_ESM.pdf]

A protocol for monitoring plant responses to changing nitrogen deposition regimes in Alberta bogs;

Monitoring and Assessment, Dale H. Vitt (dvitt@siu.edu), Melissa House, Samantha Kitchen, R. Kelman Wieder

Table S3 Mean (± S.E.) for *Picea mariana* leader length

Site	Leader length (cm)
Anzac	4.03 ± 0.33
Horse Creek	1.87 ± 0.13
JPH4	5.00 ± 0.44
Kearl	3.15 ± 0.18
MacKay	3.00 ± 0.21
McMurray	3.04 ± 0.19
